# Mass flowering of the tropical tree *Shorea beccariana* was preceded by expression changes in flowering and drought-responsive genes

**DOI:** 10.1111/mec.12344

**Published:** 2013-05-08

**Authors:** Masaki J Kobayashi, Yayoi Takeuchi, Tanaka Kenta, Tomonori Kume, Bibian Diway, Kentaro K Shimizu

**Affiliations:** * Institute of Evolutionary Biology and Environmental Studies, Institute of Plant Biology and Zurich-Basel Plant Science Center, University of ZurichCH-8057 Zurich, Switzerland; † National Institute for Environmental Studies, Center for Environmental Biology and Ecosystem StudiesTsukuba, Ibaraki 305-8506, Japan; ‡ Sugadaira Montane Research Center, University of TsukubaSugadaira-kogen, Ueda, 386-2204, Japan; § School of Forestry and Resource Conservation, National Taiwan UniversityTaipei, 10617, Taiwan; ¶ Botanical Research Centre SemenggohKm 20 Borneo Height Road, 93250 Kuching, Sarawak, Malaysia

**Keywords:** Dipterocarpaceae, flowering time, mass flowering, next-generation sequencing, *Shorea beccariana*, transcriptomics

## Abstract

Community-level mass flowering, known as general flowering, which occurs in South-East Asia at supra-annual irregular intervals, is considered a particularly spectacular phenomenon in tropical ecology. Recent studies have proposed several proximate factors inducing general flowering, such as drought and falls in minimum temperature. However, limited empirical data on the developmental and physiological processes have been available to test the significance of such factors. To overcome this limitation and test the hypotheses that general flowering is triggered by the proposed factors, we conducted an ‘ecological transcriptome’ study of a mass flowering species, *Shorea beccariana*, comparing meteorological data with genome-wide expression patterns obtained using next-generation sequencing. Among the 98 flowering-related genes identified, the homologs of a floral pathway integrator, *SbFT*, and a floral repressor, *SbSVP*, showed dramatic transcriptional changes before flowering, and their flowering functions were confirmed using transgenic *Arabidopsis thaliana*. Expression in drought-responsive and sucrose-induced genes also changed before flowering. All these expression changes occurred when the flowering-inducing level of drought was reached, as estimated using data from the preceding 10 years. These genome-wide expression data support the hypothesis that drought is a trigger for general flowering.

## Introduction

The time at which flowering occurs has a considerable effect on plants’ reproductive success. Recent molecular and genetic studies, which use a few model plants in controlled laboratory conditions, have revealed that flowering is regulated by a gene network that integrates multiple environmental and endogenous cues (Amasino 2010; Fornara *et al*. 2010). However, little genomic information is available for most plant species, which limits the study of flowering-time regulation at the molecular level in nonmodel plants. Furthermore, there have been only a few studies that have tested the molecular regulation of flowering in complex natural environments (Wilczek *et al*. 2009; Aikawa *et al*. 2010; Kobayashi & Shimizu 2011; Shimizu *et al*. 2011; Chew *et al*. 2012; Richards *et al*. 2012). Thus, it is still difficult to characterize flowering-time networks in diverse and complex environments.

Despite these difficulties, molecular approaches can be powerful tools to study flowering of nonmodel plants in naturally fluctuating environments, or ‘*in natura’* (Shimizu *et al*. 2011). Recent advances in high-throughput sequencing technologies have made it possible to analyse whole-genome transcriptome data in nonmodel organisms (Wang *et al*. 2009; Kobayashi & Shimizu 2011). In addition to technical advances, recent studies suggest that analysing whole-genome transcriptome data in natural conditions in combination with ecological and meteorological data, which has been dubbed the ‘ecological transcriptome’ approach (Richards *et al*. 2009), could facilitate understanding of how plants respond to dynamic and complex environments (Kobayashi & Shimizu 2011; Shimizu *et al*. 2011; Richards *et al*. 2012). For example, through whole-genome transcriptome analysis of *Populus tremula* during leaf development in natural conditions, Sjödin *et al*. (2008) detected sequential physiological changes, such as cell division activity, photosynthesis and secondary metabolism, associated with the cumulative sum of temperature after release from dormancy. Furthermore, quantitative transcriptional data that targets candidate flowering genes can provide more specific information about flowering. For example, the expression data of a candidate floral repressor gene *AhgFLC* (orthologous to *FLC* in *Arabidopsis thaliana*) in the perennial plant *A. halleri* across seasonally fluctuating temperatures showed that it utilizes temperature information during the preceding six weeks for flowering initiation (Aikawa *et al*. 2010). Therefore, an approach that integrates high-throughput sequencing, the ecological transcriptome and candidate flowering gene data into a well-characterized flowering network would help understanding of flowering in nonmodel plants.

**Fig. 1:**
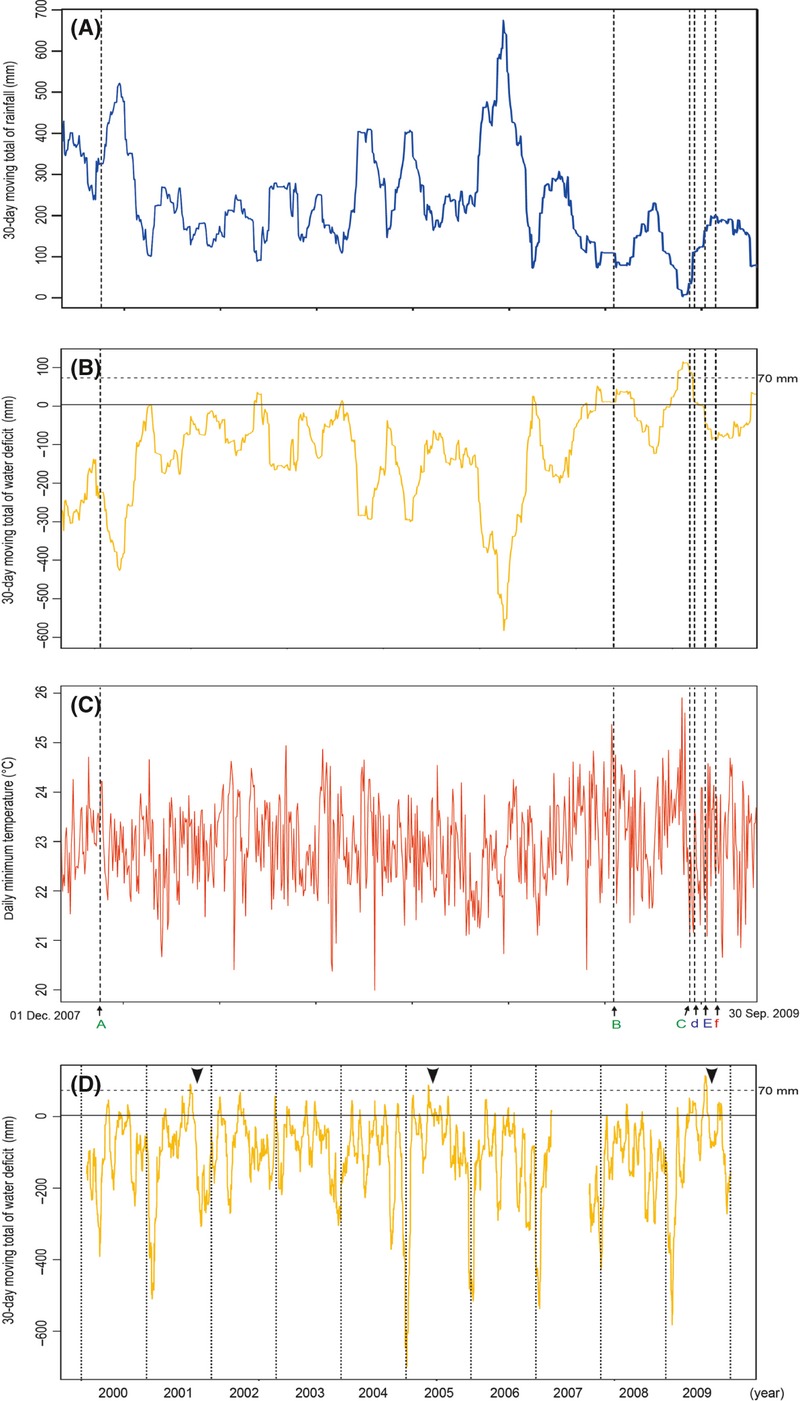
Meteorological data and occurrences of general flowering. (A)–(C) Data from December 2007 to September 2009. (A) Total rainfall over the preceding 30-day period. (B) Drought level. The dotted horizontal line indicates 70 mm. (C) Daily minimum temperature. A, B, C, d, E and f on the *x*-axis, and vertical dashed lines indicate the time points of sampling. Developmental stages of buds are vegetative buds (A to C, in green), inflorescence buds (d and E, in blue) and flower buds (f, in red), respectively. (D) Drought level and occurrences of general flowering over a 10-year period. Arrowheads indicate the general flowering events. The dotted horizontal line indicates 70 mm. The gap indicates that data were not available.

In tropical South-East Asia, some environmental factors such as rainfall, temperature and day length do not exhibit clear annual seasonality (Fig. 1; Kume *et al*. 2011), in contrast to temperate regions with a distinctive annual cycle of environmental factors. The forests in this tropical region are dominated by the family Dipterocarpaceae, which comprises more than 400 species (Ashton *et al*. 1988). These species, as well as co-occurring ones, predominantly flower synchronously at irregular intervals of less than one year to several years (Ashton *et al*. 1988; Sakai *et al*. 1999, 2006; Yasuda *et al*. 1999; Numata *et al*. 2003; Brearley *et al*. 2007). This ‘spectacular and mysterious’ phenomenon is referred to as general flowering (Sakai *et al*. 2006) and is thought to have a considerable impact not only on the reproductive success of the plant, but also on animal populations by facilitating pollination and seed predator satiation (Janzen 1974; Sakai *et al*. 1999; Kenta *et al*. 2002). In addition, general flowering has affected conservation and restoration strategies of forests because its irregular intervals make it difficult to conduct adequate seed collection (Kettle *et al*. 2010). While South-East Asia shows high species richness, it has the highest rate of deforestation of any major humid tropical region because of changes in land use (Achard *et al*. 2002; Sodhi *et al*. 2004; Hector *et al*. 2011) resulting in massive species decline and extinctions (Brook *et al*. 2003). Therefore, understanding and prediction of the irregular timing of general flowering is essential for maintaining biodiversity in this region.

Several hypotheses have been proposed to explain the regulation of the cycle of general flowering in terms of both environmental and endogenous factors. Environmental factors that occur intermittently, such as prolonged drought (Medway 1972; Appanah 1985; Sakai *et al*. 2006; Brearley *et al*. 2007), increased sunshine hours due to less cloudy conditions (Wright & Vanschaik 1994), increase or decrease in mean air temperature (Appanah 1985) and falls in minimum air temperature (Ashton *et al*. 1988; Yasuda *et al*. 1999), have been repeatedly proposed as candidate triggers for general flowering. Recent studies of long-term flowering phenology have shown that general flowering is highly correlated with drought occurrences and/or falls in minimum temperature (Ashton *et al*. 1988; Sakai *et al*. 1999, 2006; Yasuda *et al*. 1999; Numata *et al*. 2003; Brearley *et al*. 2007). Furthermore, because not all the mature individuals of a given species flower during general flowering (Ashton *et al*. 1988; Sakai *et al*. 1999), the involvement of endogenous factors in addition to environmental triggers has been discussed. Theoretical models and experimental studies have suggested that a gradual accumulation of resources over several years prior to reproduction is an important endogenous factor for regulating intermittent synchronous flowering (mass flowering) and consequent mass fruiting (e.g. Kelly & Sork 2002; Ichie & Nakagawa 2011). However, because these phenological studies and theoretical models have incorporated little empirical data on the developmental and physiological processes during flower initiation, it remains unclear how plants utilize and integrate these environmental and endogenous factors to induce general flowering.

The expression patterns of genes can provide information about the developmental and physiological processes during flower initiation. Even though the unique phenomenon of general flowering has been studied intensively in the field, little is known about which genes are functioning during general flowering due to three major obstacles. First, genomic information for species in the Dipterocarpaceae is scarce. Second, it is challenging to collect bud samples from the tropical tree canopy (∼40 m height: Kenzo *et al*. 2004), and thus, only a limited number of trees are accessible where facilities such as cranes or tree towers are available. Finally, because of the unpredictable intervals of flowering events, it is extremely difficult to observe and collect the samples immediately before floral initiation.

In 2009, mass flowering occurred for the first time since 2005 in the tropical forest within the Lambir Hills National Park in Malaysia. Due to the availability of a crane (Sakai *et al*. 2002) and continuous sampling effort, we were able to collect a time series bud samples from a single tree of *Shorea beccariana* (Dipterocarpaceae) immediately before flower initiation and during the general flowering. In this study, we focus on the hypotheses that drought and falls in minimum temperature induce flowering and test them by applying a method that integrates high-throughput sequencing, the ecological transcriptome and candidate flowering gene approaches. For this purpose, we first obtained the whole-genome transcriptome data from the time series of these unique samples, using Roche 454 next-generation sequencing. We then analysed the transcriptome data in three parts: (i) we identified the homologs to flowering-related genes and analysed their expression patterns and functions; (ii) we characterized the whole set of differentially expressed transcripts during general flowering through comparisons with 33 gene sets obtained from genome-wide transcriptome data of *A. thaliana*; and (iii) we associated the transcriptome data with meteorological data. Through this connection of molecular data and ecological responses in nature, we then discussed flowering-time control in *S. beccariana*.

## Materials and methods

### Study site

Sample collection was carried out in the Crane Plot (4 ha, 200 × 200 m; Sakai *et al*. 2002) in a lowland dipterocarp forest in the Lambir Hills National Park, Sarawak, Malaysia (4 °20′ N, 113 °50′ E, 150–250 m above sea level). The mean temperature and annual precipitation are 25.8 °C and 2600 mm, respectively, with no conspicuous annual dry season (Fig. 1A, C).

### Plant materials

Using an 85-m canopy crane, we collected three buds, each from a different branch at ∼40 m height, from an individual (ID: AA1841) of *S. beccariana* (Fig. S1) at around noon at each of six time points (TPs): 15 December 2007 (TP-A), 31 May 2009 (TP-B), 18 August 2009 (TP-C), 23 August 2009 (TP-d), 3 September 2009 (TP-E) and 14 September 2009 (TP-f) (Fig. 1). The plant materials were submerged in RNAlater (Ambion, Austin, TX, USA) immediately after harvesting and stored at −20 °C. Samples collected at TP-A, TP-B, TP-C and TP-E (denoted by upper-case letters) were used for both 454 sequencing and quantitative real-time polymerase chain reaction (qRT-PCR) experiments. Samples collected at TP-d and TP-f (denoted by lower-case letters) were used only for qRT-PCR experiments. We classified the collected buds into three categories according to their morphological characters: (i) vegetative buds; (ii) inflorescence buds; and (iii) flower buds. Morphological development during flowering is well characterized by Syamsuwida & Owens (1997) in the closely related species *Shorea stenoptera*. Vegetative buds are enclosed by two stipules between which a leaf primordium, an axillary meristem and another pair of stipules are included. This sequential structure is reiterated several times before the apex is visible. Therefore, the vegetative buds we collected contain an apex, some of the axillary meristems, stipules and leaf primordia (20–50 mg). In the vegetative stage, axillary meristems are latent in most cases, but sometimes develop into secondary and tertiary branches. At the time of floral initiation, axillary meristems develop into floral spikes, which are indeterminate inflorescences with flowers that do not have a pedicel and form compound inflorescences. Then, we refer to meristematic tissues in the early developmental stage of floral spikes that are developed from an axillary meristem as inflorescence buds (20–50 mg). The buds on the late developmental stage of floral spikes, which develop into flowers, are classified as flower buds (30–40 mg; each flower bud was halved, and one of the halves was weighed and used). The developmental stages of the collected samples were vegetative buds at TP-A, TP-B and TP-C, inflorescence buds at TP-d and TP-E and flower buds at TP-f (Fig. 1).

### 454 cDNA library construction and sequencing

We used all RNA samples described above to check the expression of selected genes in replicate samples by real-time PCR, but for genome-wide expression studies using 454, we used only one of the three samples collected from each of four time points (TP-A, TP-B, TP-C and TP-E), and total RNA extracted from each sample was used for 454 cDNA library construction. cDNA was synthesized from 1 μg of total RNA of each sample using a Super SMART™ PCR cDNA Synthesis kit (Clontech, Palo Alto, CA, USA). Following the adaptor ligation of each sample, a sequencing run was performed twice on a 454 GS FLX platform (Roche-454 Life Sciences, Branford, CT, USA) using about one-sixth of a plate for each sample (corresponding to a data volume of approximately one and a third plates in total), in accordance with the manufacturer's protocols, at the Functional Genomics Center Zurich, University of Zurich.

### Transcript assembly and sequence analysis

Sequence reads from each sample were combined and assembled into transcript contigs using newbler assembler software version 2.8 (Roche-454 Life Sciences). Adaptor sequences (Table S1) were trimmed in the process of assembly by Newbler. The contigs and singletons are hereafter referred to collectively as unigenes. All unigenes were annotated by query against the proteomes of *A. thaliana* (TAIR 10; http://www.arabidopsis.org) using BLASTx (e-value cut-off: 1.0E–10) (Altschul *et al*. 1990). To facilitate annotation of flowering genes, 161 protein-coding flowering genes of *A. thaliana* listed by Fornara *et al*. (2010) were used. *At* and *Sb* were assigned to the beginning of gene names to denote the genes of *A. thaliana* and *S. beccariana*, respectively.

### Identification of differentially expressed unigenes

The number of reads mapped to each unigene was counted for each sample. For this analysis, singletons were excluded because of their low expression levels. Furthermore, if the contigs have splicing variants, some reads are mapped to multiple splicing variants. Therefore, to avoid counting these reads redundantly, only one splicing variant was selected using the following criteria: (i) select contigs with lower BLASTx e-values; and (ii) select contigs to which more reads are mapped. The resulting read count data were used as the input for the package DEGseq (Wang *et al*. 2010) in r version 2.14.0, which identifies differentially expressed genes. For continuous transcriptome data such as microarray data measured by fluorescence intensities, a *t*-test is commonly used to detect differentially expressed genes because the abundances of transcripts across samples are approximated by a normal distribution. However, read count data do not follow a normal distribution. Therefore, procedures that are successful for continuous transcriptome data are not directly applicable to read count data. DEGseq uses a random sampling model (Jiang & Wong 2009), under which count data are assumed to follow a binomial distribution. Then, it compares the read number data between paired samples using a MA-plot-based method (Yang *et al*. 2002), where M is the log ratio of the counts between two samples for gene *g* and A is the average log counts of *g*. Because M and A are both normally distributed when samples are obtained from independent conditions, the Z-score statistic for *g* can be calculated under the null hypothesis that the probabilities of the read counts from a specific gene are the same between the samples. Then, the *P*-value, to indicate whether *g* is differentially expressed, can be calculated (see Wang *et al*. 2010 for the details of statistical methods). Unigenes were considered differentially expressed when significant expression changes (false discovery rate: *q*-value <0.001) were detected in any paired samples (TP-A and TP-B, TP-A and TP-C, TP-A and TP-E, TP-B and TP-C, TP-B and TP-E, TP-C and TP-E). The differentially expressed unigenes identified here were used for the subsequent analyses.

### Characterization of the differentially expressed unigenes through comparison with whole-genome transcriptome data of *A. thaliana*

Whole-genome transcriptome analyses using *A. thaliana* have revealed that different conditions induce and repress specific sets of genes, although some of the genes are common to multiple sets. Conversely, therefore, if significant overlapping of genes is observed in two gene sets, it could be assumed that the plants used in the experiments experienced similar conditions (e.g. Harb *et al*. 2010). To characterize the types of genes that are differentially expressed in *S. beccariana*, a set of the differentially expressed unigenes was compared with 33 publicly available gene sets of *A. thaliana* obtained by microarray analyses, and the significance of the number of overlapping genes was tested. These 33 gene sets were the lists of up- or downregulated genes responding to environmental or endogenous factors and were selected to cover a wide range of plant responses (Table 1; details of the *A. thaliana* gene sets are described in Appendix S1). To test the significance of the number of overlapping genes between *S. beccariana* and *A. thaliana*, a Fisher's exact test was performed based on the 2*2 table (Fig. S2A). After applying the Fisher's exact tests, a Bonferroni multiple testing correction (*P*-value cut-off: 0.001) was conducted.

**Table 1:** Overrepresentation of *A. thaliana* gene sets in the differentially expressed unigenes of *S. beccariana*

Environmental/endogenous factors	*A. thaliana* gene set	X1	X2	X3	X4	*P*-value
Environment (drought 1)	Upregulated genes under severe drought condition causing wilting and death	224	1347	904	6092	NS
Environment (drought 2)	Downregulated genes under severe drought condition causing wilting and death	274	1373	854	6066	NS
Environment (drought 3)	Upregulated genes under prolonged moderate drought condition under which plants survive and acclimate	31	82	1097	7357	NS
Environment (drought 4)	Downregulated genes under prolonged moderate drought condition under which plants survive and acclimate	55	125	1073	7314	2.68E–08
Environment (stress 1)	Commonly upregulated genes under 41 abiotic and biotic stress conditions (general stress response genes)	50	89	1078	7350	1.68E–10
Environment (temperature 1)	Upregulated genes with temperature decrease	74	192	1054	7247	4.06E–09
Environment (temperature 2)	Downregulated genes with temperature decrease	50	202	1078	7237	NS
Endogenous (phytohormone 1)	Upregulated genes with abscisic acid treatment	114	483	1014	6956	NS
Endogenous (phytohormone 2)	Downregulated genes with abscisic acid treatment	76	408	1052	7031	NS
Endogenous (phytohormone 3)	Upregulated genes with brassinosteroid treatment	21	75	1107	7364	NS
Endogenous (phytohormone 4)	Downregulated genes with brassinosteroid treatment	23	104	1105	7335	NS
Endogenous (phytohormone 5)	Upregulated genes with cytokinin treatment	11	61	1117	7378	NS
Endogenous (phytohormone 6)	Downregulated genes with cytokinin treatment	19	36	1109	7403	NS
Endogenous (phytohormone 7)	Upregulated genes with ethylene treatment	13	36	1115	7403	NS
Endogenous (phytohormone 8)	Downregulated genes with ethylene treatment	23	107	1105	7332	NS
Endogenous (phytohormone 9)	Upregulated genes with indole-3-acetic acid treatment	36	104	1092	7335	NS
Endogenous (phytohormone 10)	Downregulated genes with indole-3-acetic acid treatment	18	66	1110	7373	NS
Endogenous (phytohormone 11)	Upregulated genes with jasmonic acid treatment	75	239	1053	7200	4.73E–06
Endogenous (phytohormone 12)	Downregulated genes with jasmonic acid treatment	36	176	1092	7263	NS
Endogenous (phytohormone 13)	Upregulated genes with gibberellin in young flower buds	26	114	1102	7325	NS
Endogenous (phytohormone 14)	Downregulated genes with gibberellin in young flower buds	44	89	1084	7350	7.00E–08
Endogenous (nutrient 1)	Upregulated genes under carbon-limitation condition	59	188	1069	7251	NS
Endogenous (nutrient 2)	Downregulated genes under carbon-limitation condition	102	267	1026	7172	1.18E–12
Endogenous (nutrient 3)	Upregulated genes after the addition of sucrose under carbon-limitation condition	213	728	915	6711	7.17E–16
Endogenous (nutrient 4)	Downregulated genes after the addition of sucrose under carbon-limitation condition	95	520	1033	6919	NS
Endogenous (nutrient 5)	Upregulated genes under long-term phosphate limitation condition	28	117	1100	7322	NS
Endogenous (nutrient 6)	Downregulated genes under long-term phosphate limitation condition	13	42	1115	7397	NS
Endogenous (nutrient 7)	Upregulated genes under medium-term phosphate limitation condition	17	66	1111	7373	NS
Endogenous (nutrient 8)	Downregulated genes under medium-term phosphate limitation condition	0	4	1128	7435	NS
Endogenous (nutrient 9)	Upregulated genes under short-term phosphate limitation condition	11	24	1117	7415	NS
Endogenous (nutrient 10)	Downregulated genes under short-term phosphate limitation condition	1	0	1127	7439	NS
Endogenous (nutrient 11)	Upregulated genes under nitrogen limitation condition	44	151	1084	7288	NS
Endogenous (nutrient 12)	Downregulated genes under nitrogen limitation condition	22	89	1106	7350	NS

X1: The number of genes that are in both the set of differentially expressed unigenes in *S. beccariana* and the *A. thaliana* gene set.

X2: (The number of contigs that are included in the *A. thaliana* gene set) – X1.

X3: The number of differentially expressed unigenes that are not included in the *A. thaliana* gene set.

X4: (Total number of contigs) – X1 – X2 – X3 = 8567 – X1 – X2 – X3.

For details on X1 – 4, see Fig. S2A.

Fisher's exact test *P*-value cut-off: 0.001 after Bonferroni correction. NS indicates not significant.

The first seven and the last 26 gene sets in the table represent the genes responding to environmental and endogenous factors, respectively.

### Clustering of the differentially expressed unigenes

Nonhierarchical clustering of genes based on their expression patterns has been widely used in whole-genome transcriptome analyses to analyse the expression profiles of differentially expressed genes (e.g. Chalmel *et al*. 2007). Recently, Rodriguez *et al*. (2010) applied a nonhierarchical clustering algorithm, partitioning around medoid (PAM) (Kaufman & Rousseeuw 1990), to 454 sequencing data. Following this example, we employed a similar approach for our differentially expressed unigenes. See Appendix S2 for details.

### Examination of expression profiles of the enriched gene groups

For the gene sets of *A. thaliana* that showed significant overlap with the differentially expressed unigenes, a gene enrichment test was conducted in each PAM cluster using a Fisher's exact test with a Bonferroni multiple correction (*P*-value cut-off: 0.05) to test whether the enriched gene sets showed specific expression patterns (Fig. S2B). If a gene set was overrepresented in a PAM cluster, we considered that the gene set had this specific expression pattern.

For RNA extraction, transformation of *A. thaliana*, qRT-PCR, phylogenetic analyses, collection of meteorological data and estimation of water deficit, standard methods were used. The details of these methods are described in Appendix S3–8.

## Results

### 454 Sequencing, transcriptome assembly and identification of differentially expressed genes

454 Sequencing generated a total of 546 212 sequences with an average length of 251.7 bp (maximum: 998 bp, minimum: 23 bp). After removal of short and low-quality sequences, 459 700 sequences were retained. Of these, 386 212 sequences were assembled into 8567 contigs (Table S2), and 73 488 sequences remained as singletons. The average lengths of contigs and singletons were 693.6 bp (maximum: 7452 bp, minimum: 61 bp) and 267.3 bp (maximum: 998 bp, minimum: 50 bp), respectively. The average number of reads that mapped to the contigs was 42.8 (maximum: 8861, minimum: 3) (Table S2).

To annotate each unigene of *S. beccariana*, we used BLASTx against the *A. thaliana* proteome. Significant matches were found for 29 527 unigenes: 6561 contigs (∼76.6% of total contigs) and 22 966 singletons (∼31.3% of total singletons). Their average lengths were 750.3 bp (maximum: 7452 bp, minimum: 122 bp) and 313.4 bp (maximum: 551 bp, minimum: 101 bp), respectively. These 29 527 BLAST hit unigenes were mapped to 11 477 nonredundant *A. thaliana* genes, indicating that some *A. thaliana* genes were redundantly mapped by unigenes. In total, 2764 contigs were mapped to the same *A. thaliana* genes along with other contigs (Table S2). Among the 2764 contigs, 72.9% (2015) were mapped to the same regions of the subject *A. thaliana* genes with other contigs rather than to different nonoverlapping regions (Table S2). This result suggests that the contigs were mapped to the same *A. thaliana* genes because they are homologs rather than because they are partial sequences derived from different regions of the same genes by incomplete assembly. This is consistent with Dipterocarpaceae having a larger genome size than *A. thaliana* (Ohri & Kumar 1986; Schmuths *et al*. 2004) and thus possibly more genes (Hou & Lin 2009). Therefore, we treated these contigs as different genes in the following analyses.

Through the pairwise comparisons of all four samples, we identified a total of 1128 differentially expressed unigenes (Table S3).

### Differentially expressed flowering-related genes

First, we searched for homologs of flowering-related genes. Of the 29 527 unigenes that could be mapped to *A. thaliana* genes, 266 unigenes (consisting of 62 contigs and 204 singletons) showed homology to protein-coding flowering genes identified in *A. thaliana* (Fornara *et al*. 2010) (Table S4). These unigenes were mapped to 98 nonredundant *A. thaliana* flowering genes, indicating that more than 60% (98/161) of putative homologs were identified in *S. beccariana* and that some genes were redundantly mapped (Table S4).

**Fig. 2:**
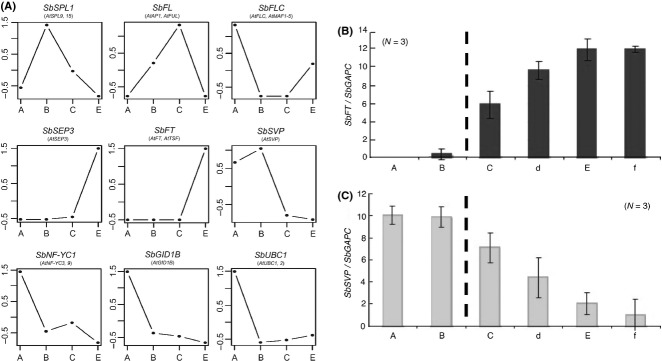
Chronological expression of the differentially expressed flowering-related genes. (A) Expression patterns obtained by 454 sequencing. The expression of each gene (*y*-axis) was scaled to a mean of 0 and a standard deviation of 1 across the four samples. A, B, C and E on the *x*-axis indicate the time points of sampling. The homologs in *A. thaliana* are indicated in parentheses. Expression patterns of (B) *SbFT* and (C) *SbSVP* by qRT-PCR. Relative transcript levels (*y*-axes) are means ± SD of three samples. The dashed lines represent the point at which the drought level reached the threshold of 70 mm. A, B, C, d, E and f on the *x*-axes indicate the time points of sampling.

Nine of these flowering-related genes were differentially expressed (Fig. 2A). By combining the results of the phylogenetic analyses, we hereafter refer to these unigenes as *SbFL*, *SbFLC*, *SbFT*, *SbGID1B*, *SbNF-YC1*, *SbSEP3*, *SbSPL1*, *SbSVP* and *SbUBC1* (Figs 2A and S3; Appendix S6).

### Validation of gene functions and expression patterns of *SbFT* and *SbSVP*

To validate the gene annotation and the expression levels of the flowering-related genes described above, we focused on two genes with contrasting functions, *SbFT* and *SbSVP*. *SbFT* is a homolog of *AtFT*, which in *A. thaliana* is a ‘floral pathway integrator’ on which flowering pathways converge and which plays a central role in flowering-time regulation (Kardailsky *et al*. 1999; Kobayashi *et al*. 1999; Fornara *et al*. 2010). *SbSVP* is a homolog of *AtSVP*, which acts as a direct upstream repressor of *AtFT* in *A. thaliana* (Lee *et al*. 2007).

**Fig. 3:**
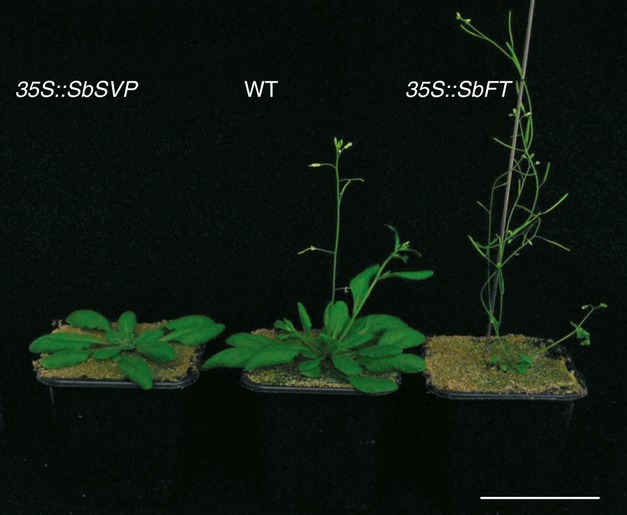
Transgenic *A. thaliana* overexpressing *SbFT* (left), *SbSVP* (right) and wild-type Columbia (middle) grown under long-day conditions for 38 days. Scale bar indicates 5 cm.

To assess whether the functions of the genes are conserved, we generated transgenic *A. thaliana* that overexpress *SbFT* or *SbSVP* under the control of the constitutive 35S promoter of cauliflower mosaic virus (Appendix S4). When compared with the wild type, the transgenic *A. thaliana* overexpressing *SbFT* showed early flowering, whereas late flowering was observed for those overexpressing *SbSVP* (Fig. 3; Table S5). These results confirmed that *SbFT* and *SbSVP* functioned as a floral promoter and repressor, respectively.

To more precisely analyse the expression patterns, qRT-PCR experiments were performed, including the additional two TPs, TP-d and TP-f (Fig. 2B, C). The expression of *SbSVP* consistently decreased from the vegetative bud stage (TP-C) until flower bud formation (TP-f). In contrast, *SbFT* expression gradually increased from the vegetative bud stage (TP-C) and was greatest in flower buds (TP-f). The expression patterns of *SbFT* and *SbSVP* quantified by qRT-PCR were thus consistent with those obtained by 454 sequencing (*SbFT*: *R*^*2*^ = 0.99, *SbSVP*: *R*^*2*^ = 0.93). In addition, qRT-PCR provided a higher resolution when the expression level was low.

These functional and expression data support the validity of expression studies in *S. beccariana* using 454 sequencing and annotation using *A. thaliana* data.

### Characterization of differentially expressed unigenes through comparisons with the whole-genome transcriptome data of *A. thaliana*

Only 9 of the 1128 differentially expressed unigenes were flowering-related genes, indicating that most of the differentially expressed unigenes have functions that are not directly related to flowering. To characterize the types of genes that are differentially expressed in *S. beccariana*, a set of the differentially expressed unigenes was compared with 33 published gene sets of *A. thaliana* obtained by microarray analyses, and the significance of the number of overlapping genes was tested. These 33 gene sets were selected to cover a wide range of plant responses to both environmental and endogenous factors. In total, significant overlapping was observed with seven of these gene sets: the sets of genes upregulated by decreasing the ambient temperature, stress conditions, jasmonic acid (JA) and increased sucrose level; and the sets of genes downregulated by prolonged moderate drought conditions, gibberellin (GA) and carbon-limitation conditions (Table 1).

### Examination of expression profiles of the enriched gene groups

**Fig. 4:**
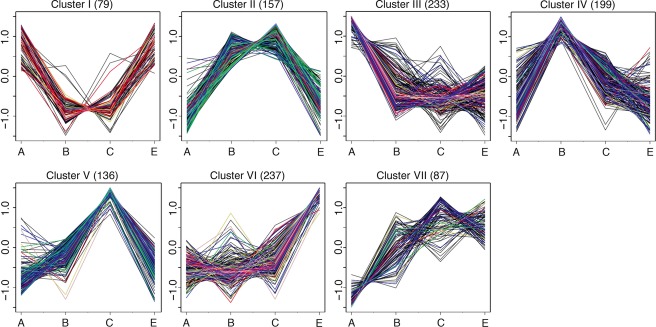
Seven clusters representing the expression patterns of the differentially expressed unigenes clustered using PAM. The numbers in parentheses represent the number of unigenes in the cluster. A, B, C and E on the *x*-axis correspond to the time points of sampling. The expression of each unigene (*y*-axis) was scaled to a mean of 0 and a standard deviation of 1 across the four samples. Each line indicates the expression pattern of each unigene. Gene categories are represented by different colours: genes upregulated by decreasing ambient temperature (green), stress conditions (yellow), JA (pink) and increased sucrose level (blue); genes downregulated by prolonged moderate drought conditions (red), GA (purple), carbon-limitation conditions (light blue) and others (black).

To analyse the timing and direction of changes in expressions of these seven gene sets, cluster analysis was first applied to all 1128 differentially expressed unigenes. The PAM clustering algorithm (Kaufman & Rousseeuw 1990) clustered the unigenes into seven clusters based on their expression patterns across the four samples (Fig. 4). Clusters I and II show inverse expression patterns with higher expression at TP-A and TP-E, and TP-B and TP-C, respectively. Clusters III, IV, V and VI show greater expression specifically at TP-A, TP-B, TP-C and TP-E, respectively. Cluster VII shows lower expression specifically at TP-A.

We then tried to identify the clusters in which the homologs of the seven gene sets are preferentially included. Six of the seven gene sets showed significant gene enrichment in a specific cluster (Tables 2 and S6). This suggests that we could detect the specific expression pattern of each of these six gene sets even though plants in the wild might be exposed to multiple signals at the same time. Importantly, overrepresentation of the homologs of downregulated genes under a prolonged moderate drought condition was observed in Cluster I, suggesting that these genes had lower expression at TP-B and TP-C than those at TP-A and TP-E (Fig. 4; Tables 2 and S6). In addition, the homologs of the genes whose expression is increased in decreasing ambient temperature were enriched in Clusters II and V and underrepresented in Cluster III (Fig. 4; Tables 2 and S6). This suggests that these genes had higher expression at TP-B and TP-C, in particular at TP-C.

**Table 2:** Over- and underrepresentation of *A. thaliana* gene sets in seven clusters based on PAM algorithm

	Cluster I Low expression at TP-B and TP-C	Cluster II High expression at TP-B and TP-C	Cluster III High expression at TP-A	Cluster IV High expression at TP-B	Cluster V High expression at TP-C	Cluster VI High expression at TP-E	Cluster VII Low expression at TP-A
Downregulated genes under prolonged moderate drought condition	**3.40E–04**	NS	NS	NS	NS	NS	NS
Commonly upregulated genes under stress conditions	**1.11E–04**	NS	NS	NS	NS	NS	NS
Upregulated genes with temperature decrease	NS	**2.45E-06**	2.02E-03	NS	**4.59E–04**	NS	NS
Upregulated with jasmonic acid treatment	NS	1.08E–02	NS	NS	3.82E-02	**7.06E–04**	NS
Downregulated genes with gibberellin in young flower buds	NS	NS	NS	NS	NS	NS	NS
Downregulated genes under carbon-limitation condition	NS	NS	NS	NS	**5.64E–04**	NS	NS
Upregulated genes after the addition of sucrose	NS	NS	NS	NS	**4.55E–03**	NS	NS

Fisher's exact test *P*-value cut-off: 0.05 after Bonferroni correction. NS represents not significant.

Over- and underrepresentation are shown in bold and normal writing, respectively.

For more details, see Fig. S2B and Table S6.

We also developed a statistical model to classify the expression pattern of each differentially expressed unigene. The best model was selected using the Akaike information criterion (AIC) **(**Akaike 1974) (see Appendix S9 for the details of the classification method). Similar results were obtained even when this classification method was used, indicating that the differences between the classification methods did not greatly affect the results of the enrichment of expression patterns (Table S7).

Furthermore, when Gene Ontology (GO) (Ashburner *et al*. 2000) was used as another annotation method to characterize the expression profiles of the differentially expressed unigenes, the same expression patterns were observed in some categories, such as the genes responding to stress and JA, although GO does not contain all the categories corresponding to 33 gene sets examined above (Fig. 4; Table S8; Appendix S10).

### Examination of meteorological data and associations with transcriptome data

The above results show that the homologs of downregulated genes under prolonged moderate drought conditions and upregulated genes in a decreasing ambient temperature have specific expression patterns. Because these factors – prolonged drought and temperature fall – have been considered as candidates for triggering general flowering (Ashton *et al*. 1988; Sakai *et al*. 1999, 2006; Yasuda *et al*. 1999; Numata *et al*. 2003; Brearley *et al*. 2007), we examined meteorological data to test whether there were changes in these factors before flowering.

Sakai *et al*. (2006) demonstrated that the flowering pattern in this forest was associated with the total rainfall during the preceding 30 days. Consistent with this observation, a dramatic reduction in total rainfall during the 30-day period was observed before the 2009 flowering event of *S. beccariana* (Fig. 1A). To investigate the relationship between drought and flowering, we calculated the total water deficit for the preceding 30 days by subtracting rainfall from the estimated amount of total evaporation (Sakai *et al*. 2006; Kume *et al*. 2011; Table S9; Appendix S8) and used it as a drought level index, in which positive values indicate drought conditions. Positive values were observed only at TP-B and TP-C (Fig. 1B; Table S9; TP-A: −227.84 mm, TP-B: 7.38 mm, TP-C: 84.52 mm, TP-E: −48.84 mm). This is consistent with the results of the transcriptome analysis, where homologs of the genes downregulated under prolonged moderate drought conditions showed lower expression levels at TP-B and TP-C (Fig. 4; Tables 2 and S6). These results suggest that *S. beccariana* was likely to be experiencing moderate drought conditions at TP-B and TP-C.

Remarkably, nondrought conditions were observed between TP-B and TP-C (Fig. 1B; Table S9), indicating that two periods of moderate drought occurred before flowering. The drought index showed that general flowering followed each of the three drought events of more than 70 mm in the preceding 10 years (Fig. 1D; Table S9), suggesting that this drought level (70 mm) might be a threshold to induce general flowering. Following this criterion, the first of the two drought events, which included TP-B, was considered to be at the drought level 1 (DL1; 0–70 mm) (Fig. 1B). The drought level 2 (DL2; >70 mm) was reached before TP-C and persisted at TP-C.

We observed a lower daily minimum temperature at TP-C (21.1 °C) than at the other three TPs (TP-A: 23.7 °C, TP-B: 24.3 °C, TP-E: 23.0 °C). The lower temperature at TP-C is reflected in the expression patterns of the upregulated genes in decreasing ambient temperature (Fig. 4; Tables 2 and S6). However, given that minimum temperature falls to less than 20 °C have been proposed as a candidate for the trigger (Yasuda *et al*. 1999), the observed minimum temperature at TP-C is not low enough to induce flowering. In addition, before the flowering event of *S. beccariana* in 2009, no significant fall in temperature to this level was observed (Fig. 1C). This suggests that the flowering in 2009 was not likely to be induced by low temperature, although the low-temperature hypothesis cannot be excluded by only one observation, and the effect of low temperature on flowering needs further investigation.

## Discussion

### Conservation and divergence of flowering-related genes

Our study identified ∼60% of the flowering-related genes of *A. thaliana* in the buds of *S. beccariana*. This is consistent with the fact that many flowering-related genes are conserved at the sequence level from herbaceous to woody species (Brunner & Nilsson 2004; Zhang *et al*. 2011) as well as phylogenetically distant species such as monocots (Higgins *et al*. 2010).

We also found that the functions and the expression patterns of many homologs of flowering-related genes were conserved in *S. beccariana*. Our results using transgenic *A. thaliana* showed that, when they were overexpressed, *SbFT* and *SbSVP* acted as a floral promoter and repressor, respectively, similarly to their homologs in *A. thaliana* (Fig. 3; Table S5). Furthermore, the expression patterns of the differentially expressed flowering genes also support the conservation of gene functions (Fig. 2A). Similar to *SbFT*, expression of *SbFL*, *SbSEP3* and *SbSPL1* was upregulated before flower initiation; these genes are the homologs of the genes that act as floral promoters (Appendix S6). Similar to *SbSVP*, expression of *SbFLC* and *SbUBC1* was downregulated before flower initiation, and these genes are the homologs of the genes that act as floral repressors.

In addition to the conserved flowering genes, it is important to consider the divergence of flowering-time regulation in *S. beccariana*. Of the 1128 differentially expressed unigenes, 160 showed no homology to any *A. thaliana* gene (Table S3). Because their expression changed dramatically before the flowering event, some of them may be involved in flowering-time regulation in *S. beccariana* with novel functions. These novel genes might explain the different flowering characteristics observed between *S. beccariana* and *A. thaliana* (i.e. differences in flowering behaviour between woody perennials and herbaceous model species or between species that flower intermittently and annually). For more similarities and differences, see Appendix S11.

### Drought as a potential trigger for flowering

The drought index and the expression patterns of the homologs responding to prolonged moderate drought conditions suggest that *S. beccariana* was likely to be experiencing moderate drought conditions at TP-B and TP-C (Figs 1B and 5; Tables 2, S6 and S9). Specifically, drought levels were at DL1 at TP-B and at DL2 of the potential flowering-inducing condition at TP-C (Fig. 1B; Table S9).

Transcriptional changes in *SbFT*, *SbSEP3* and *SbSVP* were observed only after the drought reached DL2 (Fig. 2A–C). Because *AtFT* is a floral pathway integrator playing a central role in flowering-time regulation in *A. thaliana* (Kardailsky *et al*. 1999; Kobayashi *et al*. 1999; Fornara *et al*. 2010), the induction of *SbFT* only after DL2 also supports the concept that DL2 represents a drought condition that induces general flowering. It is noteworthy that expression changes in these three genes started from the vegetative bud stage, preceding morphological changes.

Transcriptional changes in *SbSPL1*, *SbFL* and *SbFLC* were observed in both DL1 (TP-B) and DL2 (TP-C) (Fig. 2A), suggesting that expression of flowering genes in *S. beccariana* could be regulated by several steps associated with the drought level. These genes alone would not be sufficient to induce flowering because their expression had already been changed in DL1. However, they might act as upstream regulators of *SbFT*, *SbSEP3* and *SbSVP* because their expression changes started before those of *SbFT*, *SbSEP3* and *SbSVP*, and because *SbSPL1* and *SbFLC* are the homologs of upstream regulators (Searle *et al*. 2006; Kim *et al*. 2012).

Our results suggest that DL2 could be a potential trigger for general flowering. However, this study still does not provide the causal evidence. Therefore, as a next step, it is important to test the hypothesis that DL2 is a trigger for general flowering by studying whether the artificially induced DL2 drought condition causes up- and downregulation of *SbFT* and *SbSVP*, respectively, and induce flowering.

The homologs of the genes responding to a severe drought condition causing wilting and death were not enriched in the differentially expressed unigenes, suggesting that severe drought to the level of wilting might not be required for general flowering [Table 1, environment (drought 1–2)]. This suggestion is also supported by the expression patterns of the homologs of genes upregulated by stress conditions and JA (Fig. 4; Tables 2, S6 and S8). Under prolonged moderate drought conditions where plants survive and acclimate, the expression levels of genes upregulated by stress conditions and JA are repressed to even lower levels than under normal conditions as an acclimation response to continued stimulus in *A. thaliana*, whereas they are upregulated under severe drought conditions (Harb *et al*. 2010). Lower expression levels of these genes at TP-B and TP-C (Fig. 4; Tables 2, S6 and S8) are the transcriptional characteristics of moderate rather than severe drought conditions.

Global climate models have predicted more frequent El Niño-like conditions from human-induced greenhouse warming, which results in more frequent drought conditions in tropical regions (Timmermann *et al*. 1999; Neelin *et al*. 2003). Therefore, it will be important to consider the possibility that climate changes will affect the frequency of general flowering.

### Flowering and physiological states of the tree

Theoretical models and experimental studies have suggested the importance of accumulated resources for inducing flowering, which explains why not all the mature individuals flower during general flowering (e.g. Kelly & Sork 2002; Ichie & Nakagawa 2011). Our cluster analyses suggest that the homologs of the genes downregulated under carbon limitation and those upregulated after the addition of sucrose showed lower expression levels at TP-A, TP-B and TP-E and higher expression levels at TP-C, respectively (Fig. 4; Tables 2 and S6). These results may suggest that concentration of carbon in buds was limited to low levels at TP-A, TP-B and TP-E and that sugar concentration increased specifically at TP-C, when the potential flowering-inducing condition of DL2 was observed (Figs 1B and 4; Tables 2 and S6). Importantly, an increased sucrose level was not observed in the inflorescence bud (TP-E), suggesting that increased sucrose might not be used for flower development. A carbohydrate increase under flowering-inducing conditions has been reported in several species (Jones 1990; Lejeune *et al*. 1993). Furthermore, in *Sinapis alba*, a physiological model species used to study flowering, an increased sucrose supply to the meristem under inductive conditions arose from mobilization of carbohydrate reserves stored in vegetative organs (Lejeune *et al*. 1993). Our gene expression results might therefore indirectly detect a pulse of sucrose supply to the meristem from the carbohydrates stored in vegetative organs under flowering-inducing conditions. Taken in combination with the results of drought response, these findings suggest that only individuals that have accumulated a certain level of sucrose may gain competence to respond to drought to induce flowering. (For more discussion, see Appendix S12.)

## Conclusion

To our knowledge, this study is the first of its kind to demonstrate the validity of the ecological transcriptome approach in understanding the flowering-time regulation of an ecologically important nonmodel tree species. The results obtained from our unique samples suggest that drought and accumulated sucrose levels are important environmental and endogenous factors regulating flowering in *S. beccariana*, respectively, and that changes in the expression patterns of flowering genes, such as *SbFT* and *SbSVP*, occurred after the induction of flowering. Notably, their expression changes started from the vegetative bud stage, preceding morphological changes. Thus, the genome-wide expression pattern supports the hypothesis that general flowering is triggered by drought.

Due to the high cost of 454 sequencing at the commencement of this study and the difficulties in sample collection, such as limitation of canopy access, the low density of *Shorea* trees and the unpredictable intervals of flowering, this study was carried out based on a single individual tree. Further studies using more individuals will be important to elucidate more clearly the effects of environmental and endogenous factors on general flowering. Particularly, comparisons between the individuals that flower and do not flower during general flowering would tell us which factors are responsible for the different flowering responses. We hope that the identified flowering and other genes from *S. beccariana* in this study will form the basis for future studies.

The high rate of deforestation in tropical South-East Asia causes massive species decline and extinctions (Achard *et al*. 2002; Brook *et al*. 2003; Sodhi *et al*. 2004). The irregular intervals of general flowering have been a problem in planning conservation and restoration strategies for the forests because of the difficulty of adequate seed collection (Kettle *et al*. 2010). According to our results, the successive monitoring of drought level and expression levels of genes, such as *SbFT* and *SbSVP*, would help in predicting the intervals of general flowering. Through accurate prediction of the timing of general flowering, gene expression studies will enable the preparation of adequate seed collections, which will enhance biodiversity conservation and management programmes.

With the advancement in sequencing technology, the cost of sequencing will decrease in the near future. Then, the ecological transcriptome approach, which is applicable to other flowering systems, will become more important in understanding and predicting flowering in diverse ecosystems.

## Notes

M.J.K., Y.T., T.K.E. and K.K.S. conceived and designed the study; M.J.K. performed experiments and data analyses; M.J.K. and Y.T. collected the samples with T.K.E.'s and B.D.'s support; T.K.U. collected meteorological data and calculated water deficit; M.J.K. and K.K.S. wrote the article; all authors discussed the results and commented on the manuscript.

## Data accessibility

DNA sequences: the DDBJ/GenBank/EMBL database AB706328–AB706330, AB706332–AB706338. Raw sequence data: the Sequence Read Archive (SRA) SRX229125. Assembled sequences: SRA SRX229175. Information about primer and adaptor sequences used in this study is shown in Table S1. Read count data of unigenes before normalization are shown in Table S2. A summary of differentially expressed unigenes is shown in Table S3. Information about homologs of flowering-related genes is shown in Table S4. Flowering time of *A. thaliana* transgenic plants overexpressing *S. beccariana* flowering genes is shown in Table S5. Details of the results of the Fisher's exact test for the PAM clusters and the AIC-based 46 groups are shown in Table S6 and S7, respectively. Results of GO term enrichment in six clusters based on the PAM algorithm are shown in Table S8. Drought data for ten years are shown in Table S9. Details of the 33 gene sets used in this study are shown in Table S10. A summary of the genes used in phylogenetic analyses is shown in Table S11.

## Supporting information

**Fig. S1** *Shorea beccariana* during general flowering.

**Fig. S2** Schematic representation of the 2*2 tables for the Fisher's exact tests.

**Fig. S3** Neighbor-joining trees of flowering-related genes in *Shorea beccariana*.

**Fig. S4** Box plots of the expression patterns of (A) seven and (B) eight clusters of the differentially expressed unigenes based on the PAM algorithm.

**Fig. S5** Position and sequence of the conserved miRNA target site within the *SPL* gene family.

**Table S1** Primer and adaptor sequences used in this study.

**Table S2** Read count data of unigenes (contigs) before normalization.

**Table S3.** Summary of differentially expressed unigenes.

**Table S4** Homologs of flowering-related genes.

**Table S5** Flowering time of *A. thaliana* transgenic plants that overexpress *S. beccariana* flowering genes.

**Table S6** Results of the Fisher's exact test (7 clusters by PAM).

**Table S7** Results of the Fisher's exact test (46 groups of expression patterns based on AIC).

**Table S8** GO term enrichment in six clusters based on the PAM algorithm.

**Table S9** Ten years of drought data.

**Table S10** Details of the 33 A. *thaliana* gene sets used in this study.

**Appendix S1** Details of the 33 gene sets of *Arabidopsis thaliana*.

**Appendix S2** Number of clusters by PAM clustering.

**Appendix S3** RNA extraction.

**Appendix S4** Transformation experiments.

**Appendix S5** Quantitative real-time polymerase chain reaction (qRT-PCR) analysis.

**Appendix S6** Phylogenetic analyses of flowering genes.

**Appendix S7** Meteorological data collection.

**Appendix S8** Estimation of water deficit.

**Appendix S9** Examination of expression profiles of gene groups with a different clustering method based on the Akaike information criterion (AIC).

**Appendix S10** Gene Ontology (GO) term enrichment analysis.

**Appendix S11** Conservation and divergence of the regulatory network for flowering genes in *S. beccariana*.

**Appendix S12** Flowering and physiological states of the trees.
