# Supplementary figures and images for: Mass flowering of the tropical tree Shorea beccariana was preceded by expression changes in flowering and drought-responsive genes
Source: Mol Ecol. 2013 May 8;22(18):4767–82. doi: 10.1111/mec.12344 (PMC3817532; doi:10.1111/mec.12344)

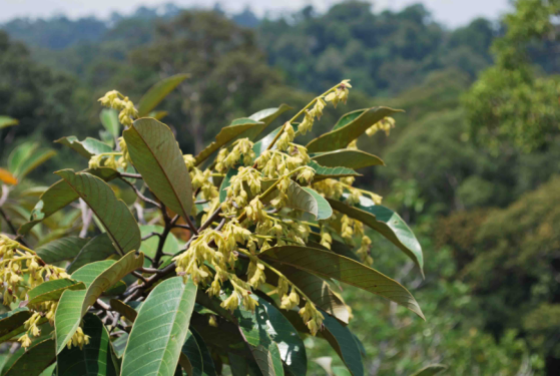

Supplement: Supplementary file 1 [file mec0022-4767-SD1.pdf]

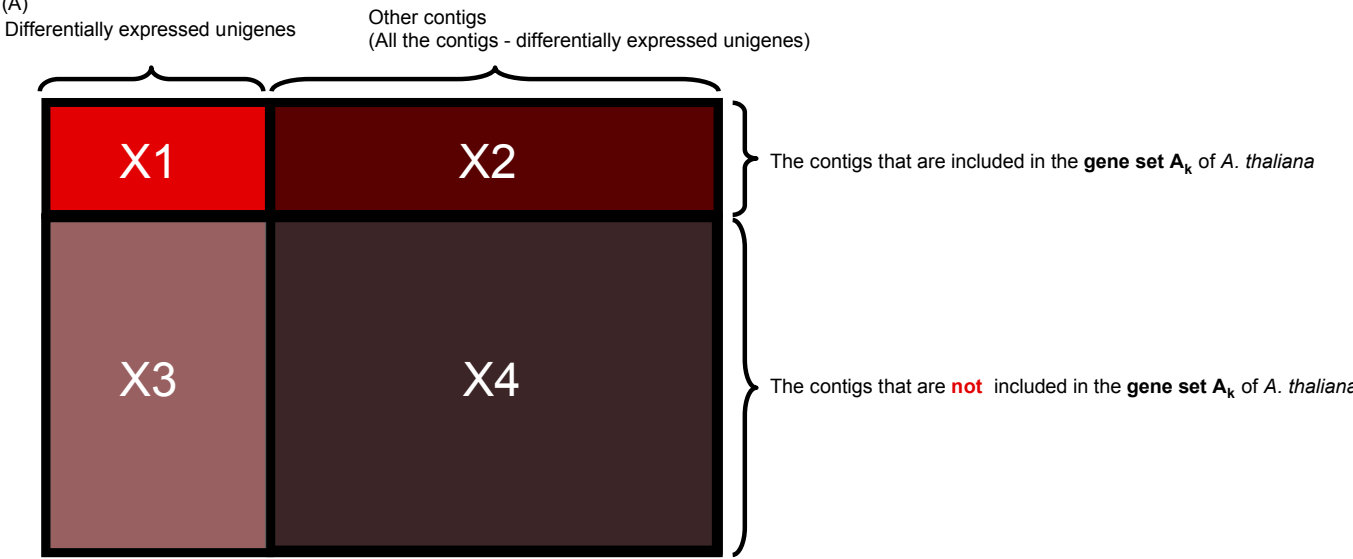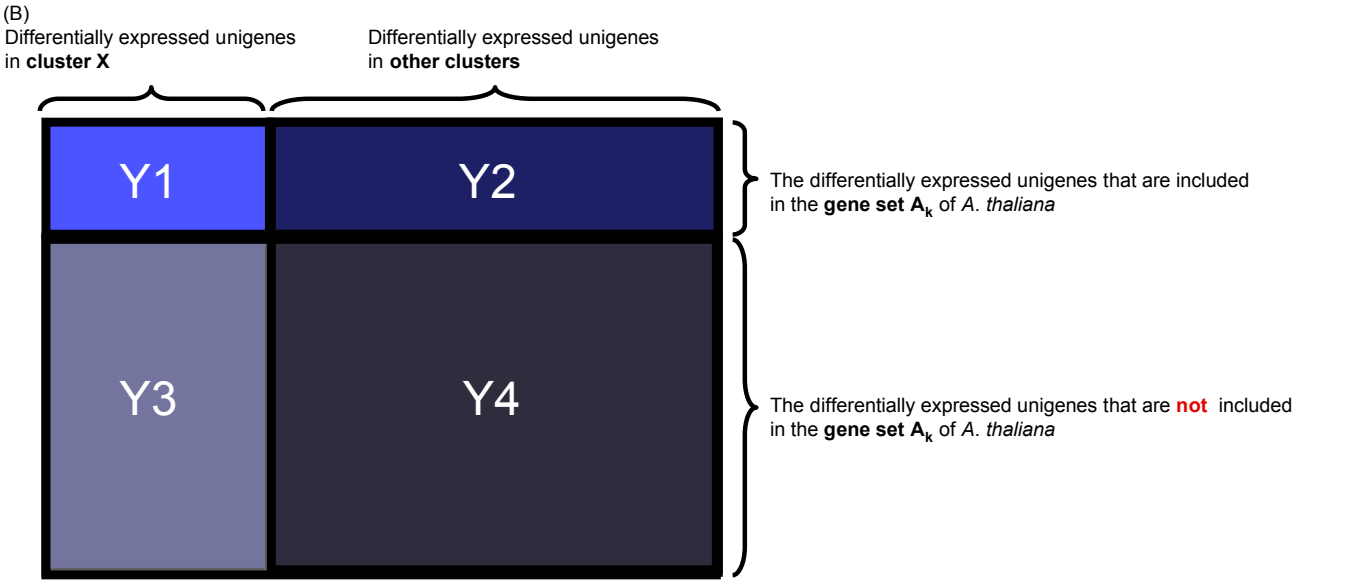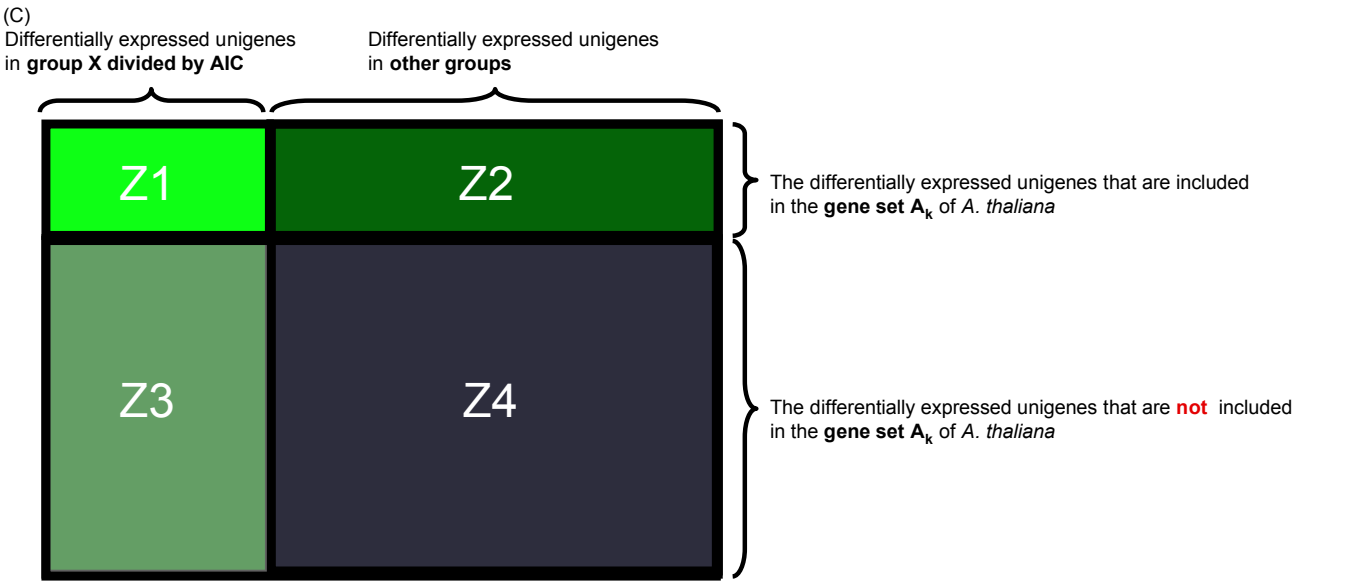

Supplement: Supplementary file 3 [file mec0022-4767-SD3.pdf]

cluster I (79)

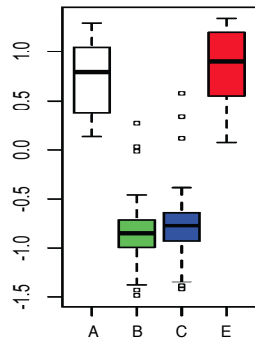

cluster II (157)

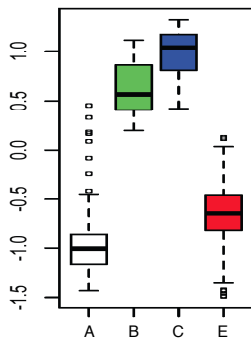

cluster III (233)

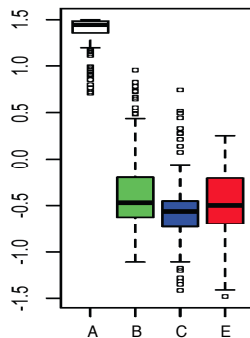

cluster IV (199)

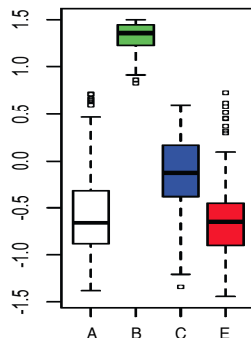

cluster V (136)

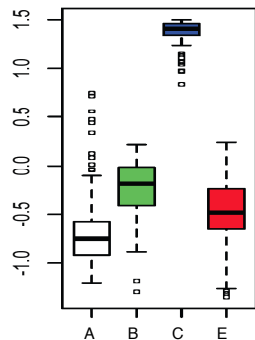

cluster VI (237)

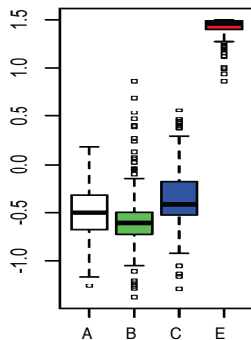

cluster VII (87)

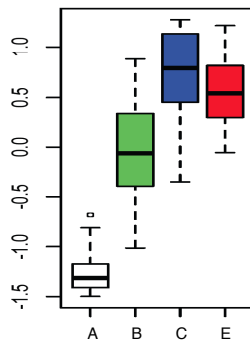

Supplement: Supplementary file 4 [file mec0022-4767-SD4.pdf]

cluster I (102)

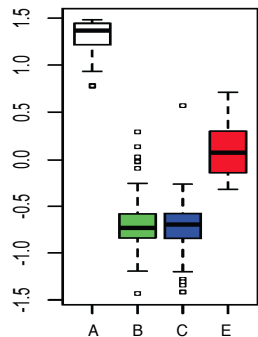

cluster II (71)

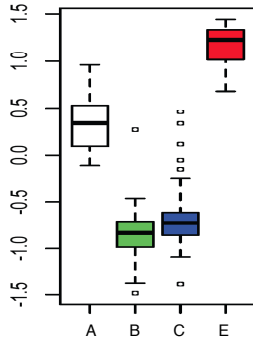

cluster III (157)

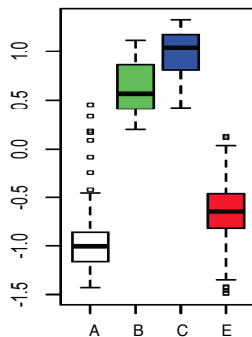

cluster IV (163)

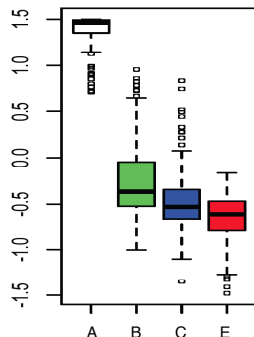

cluster V (198)

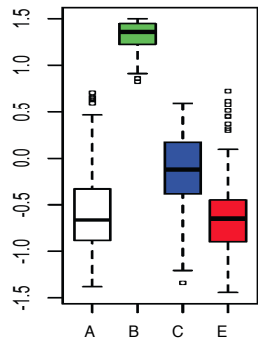

cluster VI (135)

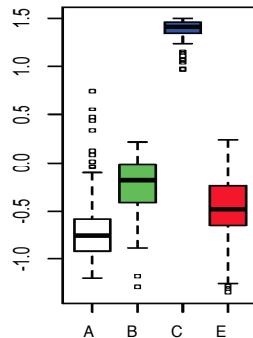

cluster VII (216)

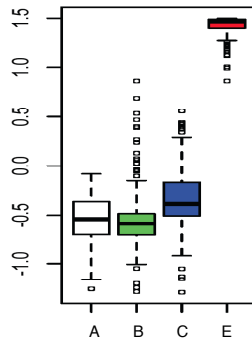

cluster VIII (86)

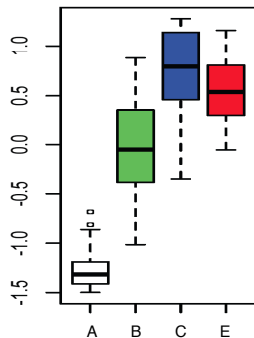

Supplement: Supplementary file 5 [file mec0022-4767-SD5.pdf]
