# Supplementary material for: Mass flowering of the tropical tree Shorea beccariana was preceded by expression changes in flowering and drought-responsive genes
Source: Mol Ecol. 2013 May 8;22(18):4767–82. doi: 10.1111/mec.12344 (PMC3817532; doi:10.1111/mec.12344)
Supplement: Supplementary file 2 [file mec0022-4767-SD2.pdf]

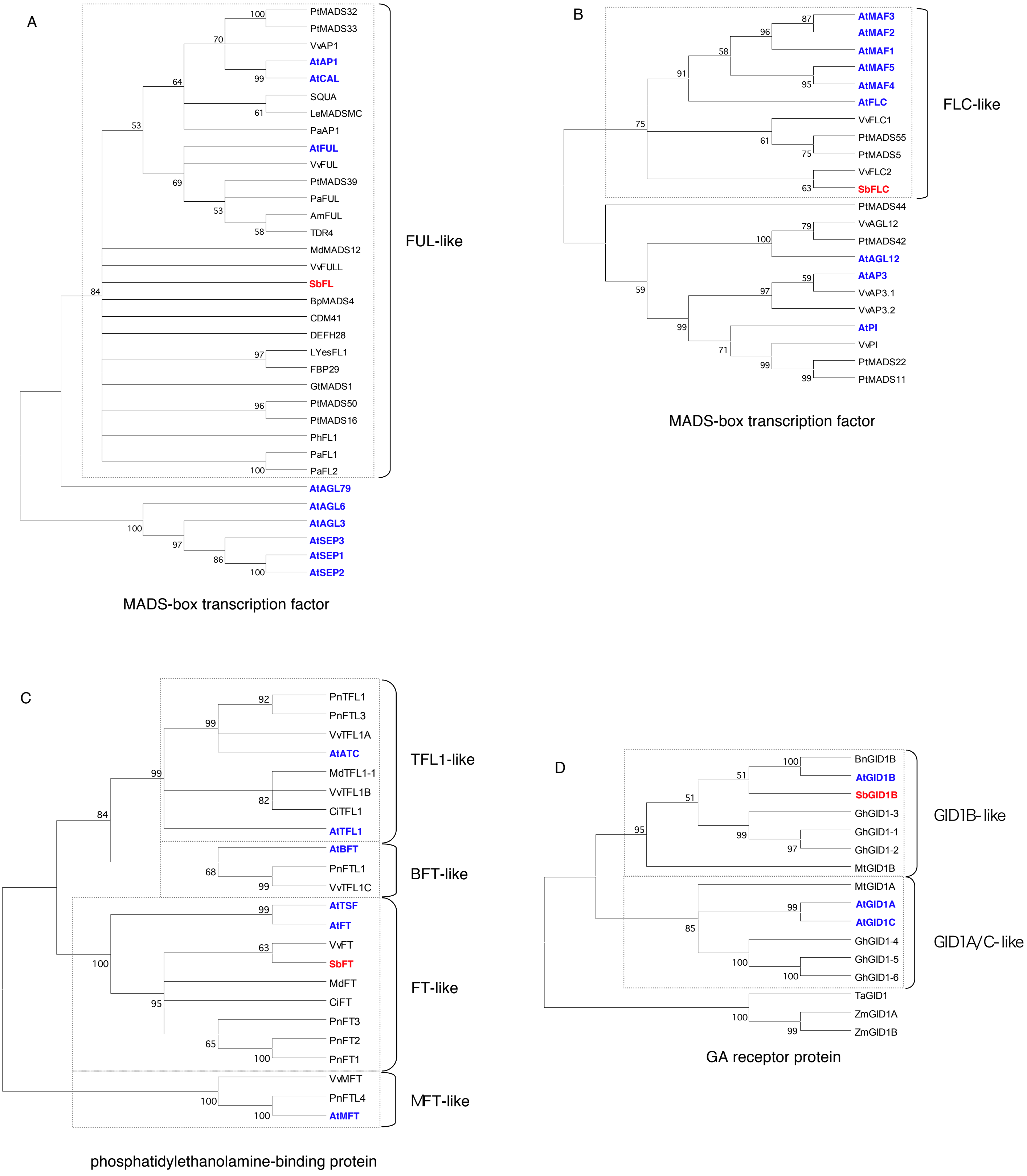
A
PtMADS32
PtMADS33
VvAP1
AtAP1
AtCAL
SQUA
LeMADSMC
PaAP1
AtFUL
VvFUL
PtMADS39
PaFUL
AmFUL
TDR4
MdMADS12
VvFULL
SbFL
BpMADS4
CDM41
DEFH28
LYesFL1
FBP29
GtMADS1
PtMADS50
PtMADS16
PhFL1
PaFL1
PaFL2
AtAGL79
AtAGL6
AtAGL3
AtSEP3
AtSEP1
AtSEP2
FUL-like
MADS-box transcription factor
B
AtMAF3
AtMAF2
AtMAF1
AtMAF5
AtMAF4
AtFLC
VvFLC1
PtMADS55
PtMADS5
VvFLC2
SbFLC
PtMADS44
VvAGL12
PtMADS42
AtAGL12
AtAP3
VvAP3.1
VvAP3.2
AtPI
VvPI
PtMADS22
PtMADS11
FLC-like
MADS-box transcription factor
C
PnTFL1
PnFTL3
VvTFL1A
AtATC
MdTFL1-1
VvTFL1B
CiTFL1
AtTFL1
AtBFT
PnFTL1
VvTFL1C
AtTSF
AtFT
VvFT
SbFT
MdFT
CiFT
PnFT3
PnFT2
PnFT1
VvMFT
PnFTL4
AtMFT
TFL1-like
BFT-like
FT-like
MFT-like
phosphatidylethanolamine-binding protein
D
BnGID1B
AtGID1B
SbGID1B
GhGID1-3
GhGID1-1
GhGID1-2
MtGID1B
MtGID1A
AtGID1A
AtGID1C
GhGID1-4
GhGID1-5
GhGID1-6
TaGID1
ZmGID1A
ZmGID1B
GID1B-like
GID1A/C-like
GA receptor protein

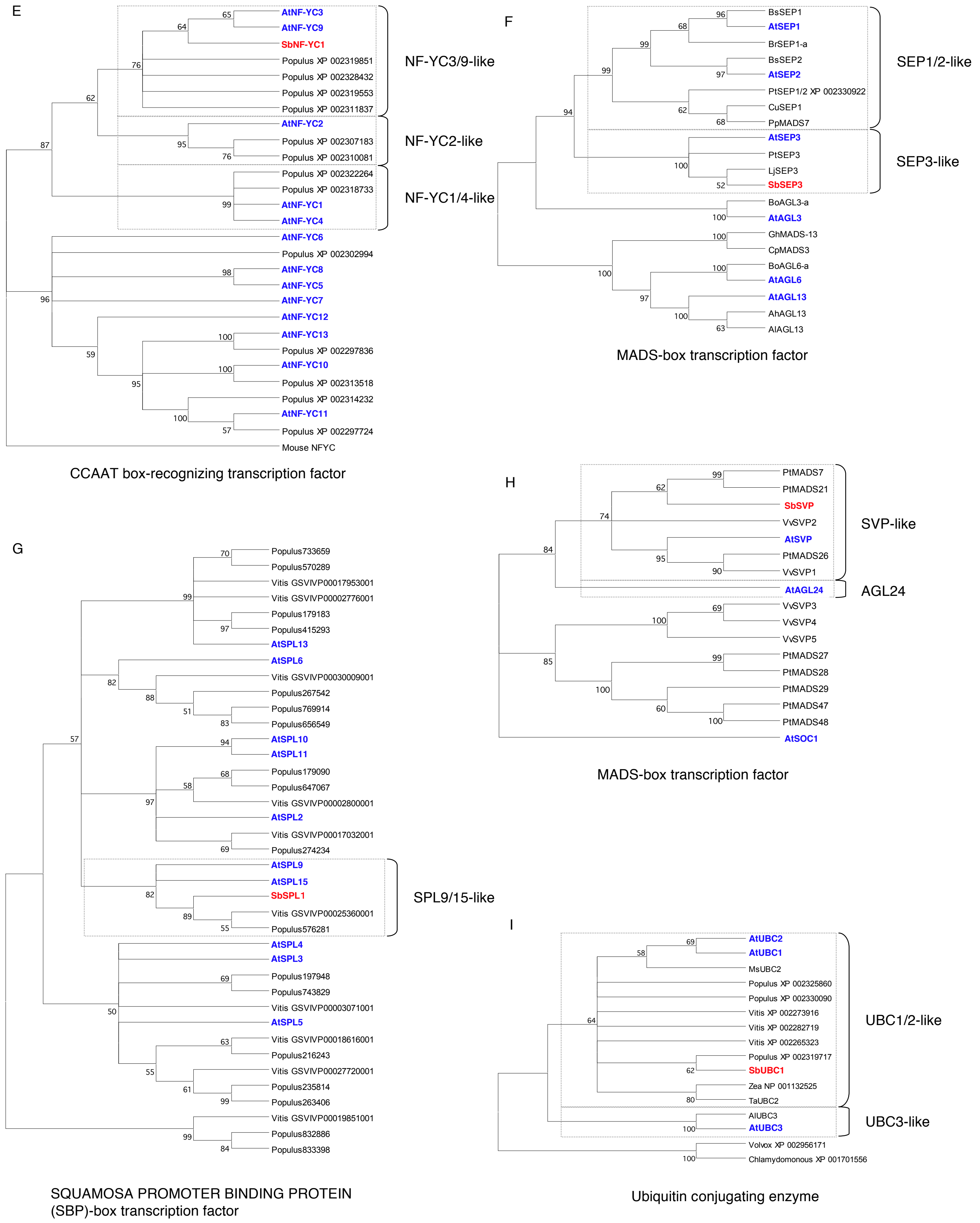
E
AtNF-YC3
AtNF-YC9
SbNF-YC1
Populus XP 002319851
Populus XP 002328432
Populus XP 002319553
Populus XP 002311837
AtNF-YC2
Populus XP 002307183
Populus XP 002310081
Populus XP 002322264
Populus XP 002318733
AtNF-YC1
AtNF-YC4
AtNF-YC6
Populus XP 002302994
AtNF-YC8
AtNF-YC5
AtNF-YC7
AtNF-YC12
AtNF-YC13
Populus XP 002297836
AtNF-YC10
Populus XP 002313518
Populus XP 002314232
AtNF-YC11
Populus XP 002297724
Mouse NFYC
NF-YC3/9-like
NF-YC2-like
NF-YC1/4-like
CCAAT box-recognizing transcription factor
F
BsSEP1
AtSEP1
BrSEP1-a
BsSEP2
AtSEP2
PtSEP1/2 XP 002330922
CuSEP1
PpMADS7
AtSEP3
PtSEP3
LjSEP3
SbSEP3
BoAGL3-a
AtAGL3
GhMADS-13
CpMADS3
BoAGL6-a
AtAGL6
AtAGL13
AhAGL13
AlAGL13
SEP1/2-like
SEP3-like
MADS-box transcription factor
G
Populus733659
Populus570289
Vitis GSVIVP00017953001
Vitis GSVIVP00002776001
Populus179183
Populus415293
AtSPL13
AtSPL6
Vitis GSVIVP00030009001
Populus267542
Populus769914
Populus656549
AtSPL10
AtSPL11
Populus179090
Populus647067
Vitis GSVIVP00002800001
AtSPL2
Vitis GSVIVP00017032001
Populus274234
AtSPL9
AtSPL15
SbSPL1
Vitis GSVIVP00025360001
Populus576281
AtSPL4
AtSPL3
Populus197948
Populus743829
Vitis GSVIVP00003071001
AtSPL5
Vitis GSVIVP00018616001
Populus216243
Vitis GSVIVP00027720001
Populus235814
Populus263406
Vitis GSVIVP00019851001
Populus832886
Populus833398
SPL9/15-like
SQUAMOSA PROMOTER BINDING PROTEIN
(SBP)-box transcription factor
H
PtMADS7
PtMADS21
SbSVP
VvSVP2
AtSVP
PtMADS26
VvSVP1
AtAGL24
VvSVP3
VvSVP4
VvSVP5
PtMADS27
PtMADS28
PtMADS29
PtMADS47
PtMADS48
AtSOC1
SVP-like
AGL24
MADS-box transcription factor
I
AtUBC2
AtUBC1
MsUBC2
Populus XP 002325860
Populus XP 002330090
Vitis XP 002273916
Vitis XP 002282719
Vitis XP 002265323
Populus XP 002319717
SbUBC1
Zea NP 001132525
TaUBC2
AlUBC3
AtUBC3
Volvox XP 002956171
Chlamydomonous XP 001701556
UBC1/2-like
UBC3-like
Ubiquitin conjugating enzyme
