# Supplementary material for: Mass flowering of the tropical tree Shorea beccariana was preceded by expression changes in flowering and drought-responsive genes
Source: Mol Ecol. 2013 May 8;22(18):4767–82. doi: 10.1111/mec.12344 (PMC3817532; doi:10.1111/mec.12344)
Supplement: Supplementary file 6 [file mec0022-4767-SD6.pdf]

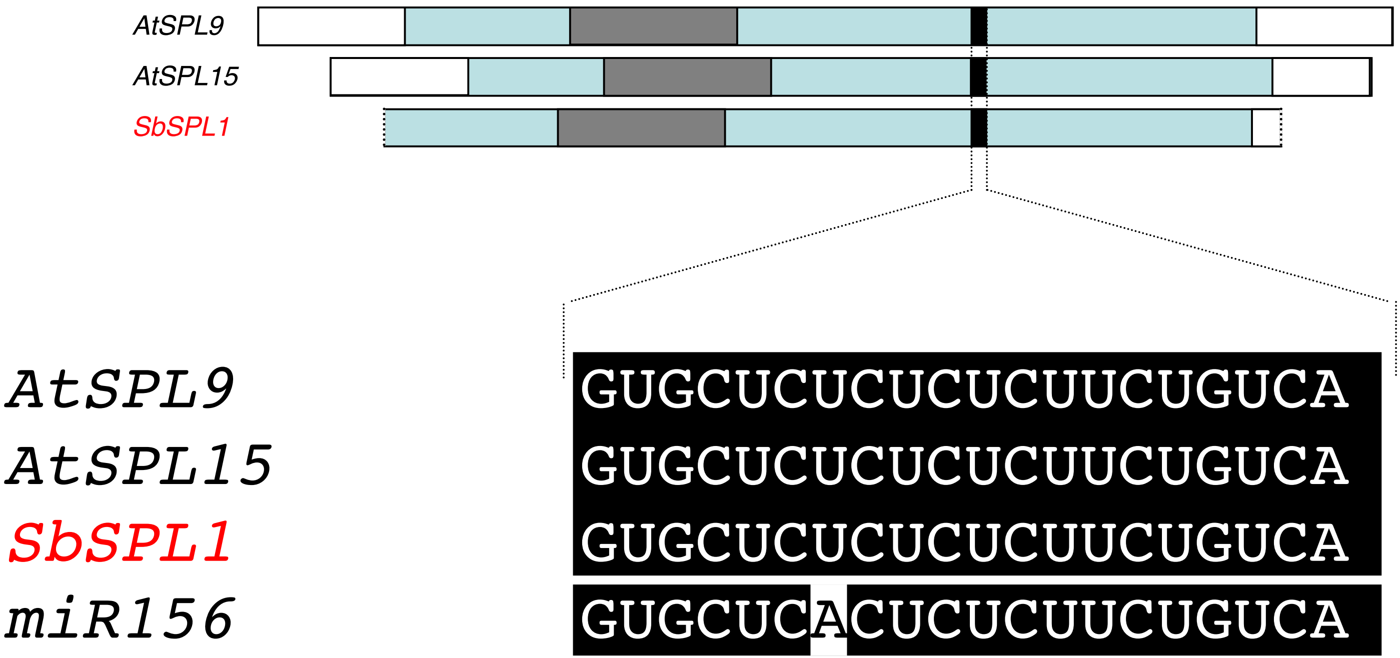
AtSPL9
AtSPL15
SbSPL1
AtSPL9
AtSPL15
SbSPL1
miR156
GUGCUCUCUCUCUUCUGUCA
GUGCUCUCUCUCUUCUGUCA
GUGCUCUCUCUCUUCUGUCA
GUGCUCACUCUCUUCUGUCA
