# Supplementary material for: Mass flowering of the tropical tree Shorea beccariana was preceded by expression changes in flowering and drought-responsive genes
Source: Mol Ecol. 2013 May 8;22(18):4767–82. doi: 10.1111/mec.12344 (PMC3817532; doi:10.1111/mec.12344)
Supplement: Supplementary file 15 [file mec0022-4767-SD15.doc]

**Supporting information**

**Mass flowering of the tropical tree *Shorea beccariana* was preceded by expression changes in flowering and drought-responsive genes**

Kobayashi *et al*.

**SI text S1. Details of the 33 gene sets of *Arabidopsis thaliana***

These 33 gene sets (Table 1) were selected to cover a wide range of plant responses to both environmental and endogenous factors. Gene sets used in this study were obtained from the papers in Table S10. When the papers contain the lists of differentially expressed genes, we used the lists directly for the comparisons. On the other hand, because gene lists were not available in the original papers for the gene sets of temperature 1 and 2 and phytohormone 13 and 14, we identified differentially expressed genes from the raw microarray data using the following procedures. Temperature1 and 2: The sets of genes up- and downregulated in response to decreasing ambient temperature were obtained by calculating the Kendall’s rank correlation coefficient between temperature and expression level from the data by Gould *et al.* (2006). If the correlation coefficient was equal to −1, the gene was considered to be an upregulated gene responding to decreasing ambient temperature; if it was equal to 1, the gene was considered to be a downregulated gene. Phytohormone 13 and 14: We identified genes up- and downregulated in response to GA from the microarray data by Cao *et al.* (2006) using *t*-test (FDR at 0.5 %).

The gene set of stress 1 obtained from Ma & Bohnert (2007) is the list of general stress response genes, which is composed of the genes commonly upregulated under 41 abiotic and biotic stress conditions. For example, the abiotic stress conditions include cold, osmotic, salt, oxidative and wounding treatments. Bacterial (*Pseudomonas syringae*) and fungal (e.g., *Botrytis cinerea*, *Erysiphe orontii* and *Phytophthora infestans*) infections are included in the biotic stress conditions.

**SI text S2. Number of clusters by PAM clustering**

First, quantile normalization, which makes the distributions identical, was performed on the count data of the differentially expressed unigenes to normalize the read numbers across the four samples. Then, the normalized read numbers of each unigene were scaled to a mean of 0 and a standard deviation of 1 across the samples. Subsequently, the resulting expression data were used for clustering. The number of clusters (k = 7) was determined by increasing the number of clusters from two to 10 until similar expression patterns were repeated in separate clusters. Here, we refer to the expression patterns that have the expression level peaks at the same time point as similar expression patterns. Until the number of clusters reached seven (k = 7), each cluster showed a different expression pattern (Fig. S4A). When the number of clusters was eight (i.e., k = 8), Clusters I and II showed similar expression patterns of the higher expression levels, specifically at TP-A and -E (Fig. S4B). Similarly, clusters with similar expression patterns were observed in the larger numbers for k. Therefore, k = 7 was used in the main text. However, we observed similar results of expression patterns of the seven gene groups in the subsequent enrichment tests even when the different cluster numbers were used, suggesting that the results of enrichment of expression patterns were not greatly affected by the number of clusters (data not shown). All the procedures of normalization and clustering were performed in R (version 2.14.0).

**SI text S3. RNA extraction**

All RNA extractions were performed at the Institute of Plant Biology, University of Zurich. First, 20–50 mg of buds were weighed, ground in CTAB buffer (3% CTAB, 1.4 M NaCl, 20 mM EDTA, 100 mM Tris–HCl pH 8.0, 0.2% b-mercaptoethanol) and incubated at 65 °C for 30 min. Following chloroform extraction, RNA was precipitated with ethanol. The resulting RNA pellet was resuspended in 50 µl of water. DNA was removed by digestion with TURBO DNA-free (Ambion, Austin, TX, USA), and RNA was purified by RNeasy column (Qiagen, Valencia, CA, USA). RNA quality was evaluated using an Agilent 2100 bioanalyzer (Agilent Technologies, Palo Alto, CA, USA).

**SI text S4. Transformation experiments**

Transgenic *A. thaliana* plants were constructed using the floral dip method (Clough & Bent 1998). Because it has been shown that *AtFT* and *AtSVP* act as a floral activator and repressor, respectively, when they are overexpressed in wild-type *A. thaliana* plants (Hanzawa *et al*. 2005, Fujiwara *et al*. 2008), we generated the transgenic plants overexpressing *SbFT* or *SbSVP* using wild-type Col to test their functions in flowering. The *SbFT* and *SbSVP* coding sequences were amplified from cDNA of an AA1841 individual using the primers listed in Table S1. The *35S::SbFT* and *35S::SbSVP* vectors were constructed by first introducing the corresponding sequences into the entry vector pCR/GW/TOPO (Invitrogen, Carlsbad, CA, USA). Then, the coding regions were transferred with LR Clonase (Invitrogen, Carlsbad, CA, USA) to destination vector pMDC32 to create the final constructs (Curtis & Grossniklaus 2003). The vectors were transformed into *Agrobacterium tumefaciens* GV3101 by electroporation. Plants in the T1 generation were used to analyze the flowering time. The number of leaves produced at bolting has been used as a common indicator of flowering time because of a close correlation observed between flowering time and number of leaves (Koornneef *et al.* 1991). Following this practice, flowering time was measured by scoring the number of primary rosette and cauline leaves. Plants were grown under long-day conditions at 22 °C.

**SI text S5. Quantitative real-time polymerase chain reaction (qRT-PCR) analysis**

cDNA was synthesized from 500 ng of total RNA of three samples at each of the six time points using a High-Capacity cDNA Reverse Transcription Kit (Applied Biosystems, Foster City, CA, USA). qRT-PCR was performed using the 7500 Fast Real-time PCR system (Applied Biosystems, Foster City, CA, USA). Primers for qRT-PCR are listed in Table S1. The *SbSVP* expression level was normalized against that of *SbGAPC* (AB706335: the DDBJ/GenBank/EMBL database) using the comparative threshold cycle method because *SbSVP* and *SbGAPC* had almost the same amplification rates. The *SbFT* expression level was normalized against that of *SbGAPC* using the standard curve method because the amplification rates of these genes were slightly different. The expression level of each gene was quantified relative to the lowest expression level of the gene. Expression levels below detection limits were treated as 0.

**SI text S6. Phylogenetic analyses of flowering genes**

Sequences of the homologs to flowering-related genes obtained by the assembly of the reads from 454 sequencing were translated into amino acid sequences, and used for phylogenetic analyses. Partial cDNA sequence of *SbSPL1* was extended by 5′ rapid amplification of cDNA ends polymerase chain reaction (RACE PCR) (Super SMART^TM^ PCR cDNA Synthesis Kit; Clontech, Palo Alto, CA, USA) and Sanger sequencing (Sang*er et a*l. 1977) using primers in Table S1 because the sequence was short. PCR products were directly sequenced using ABI PRISM 3730 Genetic Analyzer (Applied Biosystems, Foster City, CA, USA). Amino acid sequences of closely related genes were aligned using ClustalW (Thomps*on et a*l. 1994) and manually edited. Regions where the alignment was difficult were then excluded. Phylogenetic trees were generated using the neighbor-joining method as implemented in MEGA5 with 1,000 bootstrap replicates (Tamura *et al.* 2011). The sequences used in this study have been deposited in the DDBJ/GenBank/EMBL database: *SbSVP*, AB706328; *SbFT*, AB706329; *SbSPL1*, AB706330; *SbFL*, AB706332; *SbFLC*, AB706333; *SbSEP3*, AB706334; *SBUBC1*, AB706336; *SbGID1B*, AB706337; *SbNF-YC1*, AB706338. The sequences of *A. thaliana* genes were downloaded from The Arabidopsis Information Resource (TAIR 10; www.arabidopsis.org/). The other sequences used in the phylogenetic analyses are listed in Table S11.

When the clade including the gene of *Shorea beccariana* contained only one *A. thaliana* gene in the phylogenetic tree, the same gene name as in *A. thaliana* was used: *SbGID1B*, *SbSEP3* and *SbSVP*. When the clade contained some paralogs of *A. thaliana* with identifying numbers such as *SPL9* and *SPL15*, we used the identifying numbers for the homologs of *S. beccariana* starting from one: *SbNF-YC1*, *SbSPL1* and *SbUBC1*. In other cases, we followed previous studies: *SbFL* (*FRUITFUL-LIKE*): Litt & Irish (2003); *SbFLC*: Zhang *et al.* (2009); *SbFT*: Igasaki *et al.* (2008).

Phylogenetic trees indicated that: *SbFL*, *SbFLC*, *SbSEP3* and *SbSVP* are MADS-box transcription factor genes (Michaels & Amasino 1999; Sheldon *et al.* 1999; Pelaz *et al.* 2001; Lee *et al.* 2007; Melzer *et al.* 2008); *SbFT* belongs to the genes encoding phosphatidylethanolamine-binding proteins (Kardailsky *et al.* 1999; Kobayashi *et al.* 1999); *SbSPL1* is a SQUAMOSA PROMOTER-BINDING PROTEIN (SBP)-LIKE gene (Wang *et al.* 2009; Wu *et al.* 2009); and the deduced proteins encoded by *SbGID1B*, *SbNF-YC1* and *SbUBC1* are highly homologous to a GA receptor protein, a CCAAT box-recognizing transcription factor and an ubiquitin-conjugating enzyme, respectively (Griffiths *et al.* 2006; Cao *et al.* 2008; Gu *et al.* 2009; Xu *et al.* 2009; Kumimoto *et al.* 2010). Based on the literature on the phenotypes of mutants and/or transgenic plants of homologs in *A. thaliana*, *SbFL*, *SbFT*, *SbGID1B*, *SbNF-YC1*, *SbSEP3*, and *SbSPL1* were classified as putative floral promoters (Kardails*ky et a*l. 1999; Kobayas*hi et a*l. 1999; Pel*az et a*l. 2001; Griffit*hs et a*l. 2006; Melz*er et a*l. 2008; Wa*ng et a*l. 2009; *Wu et a*l. 2009; Kumimo*to et a*l. 2010), and *SbFLC*, *SbSVP*, *SbUBC1* were categorized as putative floral repressors (Michaels & Amasino 1999; Sheld*on et a*l. 1999; L*ee et a*l. 2007; C*ao et a*l. 2008; *Gu et a*l. 2009; *Xu et a*l. 2009).

**SI text S7. Meteorological data collection**

Meteorological data were collected by Kume *et al*. (2011) at the canopy crane site, which is the same site at which we collected bud samples (instruments for measuring meteorological data are 51.5 m away from the sampling tree). See Kume *et al*. (2011) for details. Radiation, wind speeds, air temperature and relative humidity were measured using data loggers every 10 min. Rainfall data were measured automatically using a tipping bucket and a data logger.

**SI text S8. Estimation of water deficit**

Water deficit was calculated daily by subtracting rainfall from total evaporation (ET) following the method of Kume *et al*. (2011). ET is the sum of transpiration (Et) and rainfall interception (Ei). Et and Ei were estimated based on the simplified big-leaf model of Kume *et al.* (2011), which was modified from the Penman–Monteith equation (e.g., Monteith & Unsworth 1990). The mean values of Et and Ei were calculated from the estimated maximum and minimum of Et and Ei, respectively. These mean values were then used in calculating the water deficit.

**SI text S9. Examination of expression profiles of gene groups with a different clustering method based on the Akaike information criterion (AIC)**

**In combination with the results of clustering by the PAM algorithm and enrichment test, we obtained the characteristic expression patterns of** the homologs of seven gene groups (i.e., the sets of genes upregulated by decreasing the ambient temperature, stress conditions, jasmonic acid (JA) and increased sucrose level, and the sets of genes downregulated by prolonged moderate drought condition, GA and carbon-limitation conditions (Table 1). To test whether these expression patterns are independent of clustering methods, clustering by another method based on the Akaike information criterion (AIC) (Akaike 1974) and enrichment tests were conducted.

## **9-1. AIC-based classification of expression patterns of the differentially expressed unigenes**

In this section, we describe how the expression pattern of each unigene was determined by model selection using AIC. We first considered possible models representing the expression patterns of each differentially expressed unigene. When the four samples (TP-A, -B, -C, and -E) are classified by whether they have the same expression levels or not, they can be classified into 15 groups. The possible 15 groups are represented as follows, using brackets grouping the samples with the same expression level. All the samples were classified into the same group (i.e., all the samples have the same expression level): 1: [(TP-A, -B, -C, -E)]. The samples were classified into two groups with different expression levels: 2: [(TP-A, -B, -C)(TP-E)]; 3: [(TP-A, -B, -E)(TP-C)]; 4: [(TP-A, -C, -E)(TP-B)]; 5: [(TP-B, -C, -E)(TP-A)]; 6: [(TP-A, -B)(TP-C, -E)]; 7: [(TP-A, -C)(TP-B, -E)]; 8: [(TP-A, -E)(TP-B, -C)]. The samples were classified into three groups with different expression levels: 9: [(TP-A, -B)(TP-C)(TP-E)]; 10: [(TP-A, -C)(TP-B)(TP-E)]; 11: [(TP-A, -E)(TP-B)(TP-C)]; 12: [(TP-B, -C)(TP-A)(TP-E)]; 13: [(TP-B, -E)(TP-A)(TP-E)]; 14: [(TP-C, -E)(TP-A)(TP-B)]. The samples were classified into four groups with different expression levels: 15: [(TP-A)(TP-B)(TP-C)(TP-E)]. In each group, we assumed that the read numbers of the samples followed a normal distribution, and thus that the samples in the same brackets (e.g., (TP-A, -B)) had the same mean and standard deviation values of the read number. Then, these 15 groups were considered as models to be compared. Because the number of parameters to be estimated is two (i.e., mean and standard deviation) in each bracket, the total number of parameters is calculated by multiplying the number of brackets by two in each model. If the brackets contained only one sample, the standard deviation was assumed to have the same value as the samples in other brackets. In the case of group 15, a standard deviation of combination 1 was used. Using these means and standard deviations, the likelihood of each model was calculated. We then calculated the AIC value in each model following the definition (–2log(likelihood)+2(number of parameters)) (Akaike 1974), and chose the model with the lowest AIC as the expression pattern of the unigene. Each model can further be classified into different groups comparing the expression levels of subgroups in brackets (e.g., the model 2, [(TP-A, -B, -C)(TP-E)], can be classified into two groups, [(TP-A, -B, -C) < (TP-E)] or [(TP-E) < (TP-A, -B, -C)], depending on the expression levels of the subgroups). Although 75 groups of expression patterns are theoretically possible (i.e., model 1: one expression pattern, models 2–8: two expression patterns, models 9–14: six expression patterns, model 15: 24 expression patterns), 29 groups of expression patterns did not contain any unigenes. Therefore, the expression patterns of the differentially expressed unigenes were finally classified into 46 different groups (Table S7).

## **9-2. Enrichment of *A. thaliana* gene sets in AIC-based groups**

For the seven gene sets of *A. thaliana* that showed a significant overlap with the set of differentially expressed unigenes, a gene enrichment test was conducted in each AIC-based group by a Fisher’s exact test based on a 2*2 table to test whether the enriched genes showed specific expression patterns (Fig. S2C). A *P*-value of less than 0.05 was considered a significant over- or underrepresentation of gene sets.

Table S7 shows the results of the enrichment tests. These results were similar to those obtained by clustering with a PAM algorithm, indicating that the suggested expression patterns were not greatly affected by clustering methods.

**SI text S10. Gene Ontology (GO) term enrichment analysis**

In addition to the comparisons with 33 gene sets of *A. thaliana*, we used Gene Ontology (GO) (Ashburn*er et a*l. 2000) as an annotation method to characterize the expression profiles of the differentially expressed unigenes.

GO annotations of *A. thaliana* were downloaded from TAIR 10 (www.arabidopsis.org/). GO term enrichment among the differentially expressed unigenes in PAM clusters was performed using the “elim” algorithm in the topGO package in R (Ale*xa et a*l. 2006). When a GO term is significantly enriched, more general (higher levels of) GO terms also tend to show significant enrichment because of the structure of GO. To avoid this redundancy, the “elim” algorithm considers GO graph topology and removes the genes mapped to significant GO terms from more general ones. To test the significance of the enrichment, Fisher’s exact test was used (*P*-value cutoff: 1.0E–04).

Table S8 shows the results of the GO term enrichment of biological processes (BP) and molecular functions (MF). We observed the enrichment of “response to wounding” and “defense response to fungus” for BP in Clusters III and VI, respectively (Fig. 4; Table S8). These GO terms are child terms (more specific terms) of “response to stress,” suggesting that the homologs of the genes responding to stress showed lower expression levels at TP-B and -C. Furthermore, although *P*-values are not less than 1.0E–04, we observed the enrichment of “response to JA stimulus” for BP in Cluster I (*P*-value: 1.12E–03) and Cluster VI (*P*-value: 3.71E–03), suggesting that the homologs of the genes responding to JA stimulus showed lower expression levels at TP-B and -C (Fig. 4; Table S8). These results are consistent with the results obtained by comparisons of *A. thaliana* transcriptome data such that the genes upregulated by several stress conditions and JA commonly had lower expression levels at TP-B and -C (Fig. 4; Tables 2 and S6).

Focusing on the drought conditions at TP-B and -C, we observed enrichment of the GO terms relevant to protein synthesis, including the “structural constituent of ribosome” for MF and a “translation” and “ribosome biogenesis” for BP in Clusters II and V and “translational elongation” for BP in Cluster V (Fig. 4; Table S8). Furthermore, we observed the enrichment of “response to heat”, “response to high light intensity”, “response to hydrogen peroxide” and “heat acclimation” for BP in Clusters III and VI (Fig. 4; Table S8). Further examination showed that most of these genes (47 out of 66) encoded heat shock proteins, which are the proteins involved in the folding and unfolding of other proteins. Because the folding of proteins is an important process after translation, these results may suggest that protein synthesis was activated during the drought conditions at TP-B and -C.

In Cluster VI, we observed enrichment of “sequence-specific DNA binding transcription factor activity” for MF and “regulation of transcription, DNA-dependent” for BP, suggesting that the genes responsible for transcription showed higher expression levels at TP-E (Fig. 4; Table S8). As the buds at TP-E were already developed into inflorescence buds, these results may suggest that transcriptional activity was elevated, associated with morphological changes.

Although annotation by GO terms is systematic, we cannot examine the categories that are not included in GO terms, such as “response to prolonged moderate drought” and “response to ambient temperature.” Therefore, annotation by comparisons with *A. thaliana* transcriptome data has advantages in characterizing the genes in such categories even though we must select the transcriptome data ourselves.

To avoid the effect of different annotation by different data sets, we used Blast2GO version 2.5.1 (http://www.blast2go.de; Conesa *et al*. 2005) to obtain GO annotation from various species. First, sequences were searched against an NCBI nonredundant (nr) protein database, which contains sequences from not only *A. thaliana*, but also other species including tree species such as *Populus trichocarpa* and *Vitis vinifera* using Blastx (e-value cutoff of 1.0E–10). Then, GO terms were assigned based on the results of Blastx. In the enrichment test, overrepresentation was not observed for the GO terms “response to chitin” in Clusters I and VI, “response to salicylic acid stimulus” in Cluster VI, and “copper ion binding” in Cluster VII, which were enriched when *A. thaliana* GO data were used. However, the other 17 GO terms that were enriched using *A. thaliana* GO data were also overrepresented in the respective clusters in this analysis, suggesting that the differences of data sets used for annotation did not greatly affect the results of enrichment tests.

**SI text S11. Conservation and divergence of the regulatory network for flowering genes in *S. beccariana***

The expression patterns of flowering genes, such as the inverse expression patterns of *SbSVP* and *SbFT*/*SbSEP3*, suggest that the regulatory network of expression of homologs to flowering genes may also be conserved in *S. beccariana* (Fig. 2A–C). In *A. thaliana*, *AtSVP* negatively regulates *AtFT* and *AtSEP3*, and their expression is gradually increased by downregulation of *AtSVP* (Lee *et al.* 2007). Similarly, expression of *SbFT* and *SbSEP3* was elevated after expression of *SbSVP* was downregulated (Fig. 2A–C). As a next example, AtUBC1 and AtUBC2 mediate histone H2B monoubiquitination and upregulate the expression of *FLC* and some of the *MAF* genes (C*ao et a*l. 2008; *Gu et a*l. 2009; *Xu et a*l. 2009). Consistent with the regulation observed in *A. thaliana*, the expression of *SbUBC1* declined in drought conditions in a manner similar to that of *SbFLC* (Fig. 2A). In addition, *SbFL* showed a similar expression pattern to that of *SbSPL1*. Transcriptional regulation of *FUL*-like genes by the SPL family of transcription factors has been shown in some species (Yamaguchi *et al.* 2009; Preston & Hileman 2010). These data may also suggest the conservation of transcriptional regulation between *SbSPL1* and *SbFL* in *S. beccariana*. In addition, *AtSPL9* and *AtSPL15* are miR156-targeted *SPL* genes in *A. thaliana* (Schw*ab et a*l. 2005; Wa*ng et a*l. 2009; *Wu et a*l. 2009). *SbSPL1* shared the miRNA target site that is a complementary sequence to miR156 (Rhoad*es et a*l. 2002) in the coding region of *SbSPL1*, located in the conserved positions of the corresponding *A. thaliana SPL* genes, *AtSPL9* and *AtSPL15* (Fig. S5). These results may imply the conservation of the miR156/*SPL* regulatory pathway, although our transcriptome data using 454 sequencing do not contain information on expression levels of miRNAs. Recently, conservation of the miR156/*SPL* regulatory pathway has been shown in the tree species *Populus canadensis* (Wa*ng et a*l. 2011).

**SI text S12. Flowering and physiological states of the trees**

Using one of the species in Dipterocarpaceae, Ichie & Nakagawa (2011) recently suggested that long-term accumulation of phosphorus could be an important factor affecting reproduction. They observed that phosphorus concentration specifically decreased in vegetative organs during reproduction. However, during the period of our experiment, our transcriptome analyses did not detect any trend of transcriptional change in the homologs in response to phosphate limitation (Table 1). This might be because of lower expression levels of these genes than the detectable level obtained by 454 sequencing. Alternatively, because our study only focused on the flowering-inducing stage, the differences between these two studies may suggest that phosphorous is required in the later stages of reproduction such as during development of reproductive organs, rather than in flowering induction. Further analyses of transcriptome data with a higher detection sensitivity and within a longer time period will elucidate the effect of the phosphate level upon reproduction.

Furthermore, the homologs responding to GA were enriched in the differentially expressed unigenes (Table 1). We also observed that *SbGID1B*, which belongs to the group of genes encoding a GA receptor protein (Griffiths *et al*. 2006), changed its expression as one of the homologs of flowering-related genes (Fig. 2A). These results might suggest that GA is involved in flowering regulation in *S. beccariana*, as reported in other plant species (e.g., Wilson *et al.* 1992; Eysteinsson & Greenwood 1995). However, it is not apparent when, if at all, GA is activated because the homologs responding to GA did not show specific expression patterns in the clustering analysis (Tables 2 and S6).

**Supporting figures**

**Figure legends**

**Figure S1** *Shorea beccariana* during general flowering.

**Figure** **S2** Schematic representation of the 2*2 tables for the Fisher’s exact tests. (A) The 2*2 table to test whether the 33 *A. thaliana* gene sets are enriched in the differentially expressed unigenes of *S. beccariana*. The differentially expressed unigenes are the subset of all the contigs. (See the method of identification of differentially expressed unigenes by DEGseq.)

X1: The number of genes that are in both the set of differentially expressed unigenes in *S. beccariana* and the gene set A_k_ of *A. thaliana.*

X2: The number of contigs that are in the gene set A_k_ of *A. thaliana* – X1.

X3: The number of the differentially expressed unigenes that are not included in the gene set A_k_ of *A. thaliana.*

X4: (Total number of contigs) – X1 – X2 – X3 = 8,567 – X1 – X2 – X3.

Gene set A_k_: 33 different gene sets (gene sets A_1_ to A_33_).

For each different gene set A_k_, a Fisher’s exact test was performed based on the 2*2 table consisting of X1 to X4.

(B) The 2*2 table to test whether the seven *A. thaliana* gene sets are over- or underrepresented in seven clusters by the PAM algorithm.

Y1: The number of genes that are in both the set of differentially expressed unigenes in Cluster X and the gene set A_k_ of *A. thaliana.*

Y2: The number of differentially expressed unigenes that are included in the gene set A_k_ of *A. thaliana*) – Y1.

Y3: The number of differentially expressed unigenes that are not included in the gene set A_k_ of *A. thaliana.*

Y4: (Total number of the differentially expressed unigenes) – Y1 – Y2 – Y3 = 1,128 – Y1 – Y2 – Y3.

Gene set A_k_: Seven different gene sets (gene sets A_1_ to A_7_)

Cluster X: Clusters I to VII by the PAM algorithm

For each Cluster X and gene set A_k_, a Fisher’s exact test was performed based on the 2*2 table consisting of Y1 to Y4.

(C) The 2*2 table to test whether the seven *A. thaliana* gene sets are over- or underrepresented in 46 groups based on AIC.

Z1: The number of genes that are in both the set of differentially expressed unigenes in group X clustered based on AIC and the gene set A_k_ of *A. thaliana.*

Z2: (The number of the differentially expressed unigenes that are included in the gene set A_k_ of *A. thaliana*) – Z1.

Z3: The number of the differentially expressed unigenes that are not included in the gene set A_k_ of *A. thaliana.*

Z4: Total number of differentially expressed unigenes – Z1 – Z2 – Z3 = 1,128 – Z1 – Z2 – Z3.

Gene set A_k_: Seven different gene sets (gene sets A_1_ to A_7_).

Group X divided by AIC: 46 groups based on AIC.

For each group X and gene set A_k_, a Fisher’s exact test was performed based on the 2*2 table consisting of Z1 to Z4.

**Figure** **S3** Neighbor-joining trees of flowering-related genes in *Shorea beccariana*. The trees were constructed using amino acid sequences. Branches with less than 50% bootstrap support were collapsed. Genes of *A. thaliana* and *S. beccariana* are in blue and red, respectively. The genes used in the phylogenetic analyses are listed in Table S11. Names of the clades containing the genes of *S. beccariana* are indicated on the right side of the clades enclosed in dotted boxes. The encoded protein is shown at the base of each tree.

**Figure S4** Box plots of the expression patterns of (A) seven and (B) eight clusters of the differentially expressed unigenes based on the PAM algorithm*.* The numbers in parentheses represent the number of unigenes in the cluster. A, B, C and E on the *x*-axis correspond to the time points of sampling (TP-A, -B, -C and -E). The *y*-axis indicates normalized expression values. The expression of each unigene was scaled to a mean of 0 and a standard deviation of 1 across four samples used for 454 sequencing.

**Figure S5** Position and sequence of the conserved miRNA target site within the *SPL* gene family. The cDNAs of the *SPL* gene family members carrying the conserved miRNA target site (black boxes) are schematically represented. Coding sequences are shaded light blue; the conserved SBP box is gray. White indicates the 5′ and 3′ UTRs. The dotted lines on *SbSPL1* indicate that the 5′ and 3′ regions of the gene have not been determined. Alignment of the sequences of the conserved miRNA target site within the *SPL* mRNAs is shown below. To allow direct comparison with miR156 of *A. thaliana*, the reverse complementary sequence of the miRNA is included. Conserved nucleotides are indicated by black shading.

**Supporting tables**

**Table S1** Primer and adaptor sequences used in this study

| Primer or adaptor name | Internal ID | Primer or adaptor sequence |
| --- | --- | --- |
| *SbFT* cloning forward primer | 1855SbFT5cloning | ATGCCCAGAGATAGATTAGATCCTC |
| *SbFT* cloning reverse primer | 1857SbFT3cloning | TTATCCTCTCCGTCCACCGGTCCCA |
| *SbSVP* cloning forward primer | 2308SbSVP_5cloning | ATGGCTCGGGAGAAGATCAAG |
| *SbSVP* cloning reverse primer | 2309SbSVP_3cloning | CTAGTGAAGGGAAAGCCCTAATTTGA |
| *SbFT* qRT-PCR forward primer | 1837SbFTRTP5 | TGCATTGGTTGGTAACTGATATTCC |
| *SbFT* qRT-PCR reverse primer | 1838SbFTRTP3 | CCCGGATTTTCATAACACACAA |
| *SbSVP* qRT-PCR forward primer | 2552SbSVPRTP5_1 | GCAGCAGCTTGAGAAAATGCT |
| *SbSVP* qRT-PCR reverse primer | 2553SbSVPRTP3_1 | TCCGTTCACCCTTGGTTTCA |
| *SbGAPC* qRT-PCR forward primer | 2560SbGAPCRTP5_1 | TGGCGTGTTCACCGACAA |
| *SbGAPC* qRT-PCR reverse primer | 2561SbGAPCRTP3_1 | CCTTCTTAGCACCACCCTTCAA |
| 5′ RACE forward primer | 1802RACEsmart5 | TGGTATCAACGCAGAGTACGCG |
| 5′ RACE for *SbSPL1* 1st reverse primer | 2691SPL9_3U1 | TGGTAATCATAAGCAAACAGCA |
| 5′ RACE for *SbSPL1* 2nd reverse primer | 2692SPL9_3U2 | AGCAAACAGCAAGGCACATA |
| *SbSPL1* sequence primer 1 | 2693SPL9_3U3 | CACATATTGCTTAAAGGGACCA |
| *SbSPL1* sequence primer 2 | 2694SPL9_3U4 | CTCGGTTGGTCTTGATGCTG |
| SMART 5′ adaptor | – | AAGCAGTGGTATCAACGCAGAGTACGCGGG |
| SMART 3′ adaptor |  | AAGCAGTGGTATCAACGCAGAGTACT |

**Table S5** Flowering time of *A. thaliana* transgenic plants that overexpress *S. beccariana* flowering genes

|  | *35S::SbFT* | WT (Col) | *35S::SbSVP* |
| --- | --- | --- | --- |
| Number of individuals | 34 | 27 | 34 |
| Number of rosette leaves | 3.2 ± 0.6 ** | 7.7 ± 0.8 | 17.8 ± 2.0 ** |
| Number of cauline leaves | 2.1 ± 0.6 * | 2.5 ± 0.6 | 3.4 ± 0.7 ** |
| Number of total leaves | 5.4 ± 0.7 ** | 10.3 ± 0.9 | 21.2 ± 2.5 ** |
| Range of number of total leaves | 4–7 | 9–12 | 17–27 |

Flowering time is recorded as mean leaf number at flowering ± SE.

Statistically significant differences (Student’s *t*-test) between transgenic plants and the wild type are indicated by * for *P* < 0.05 and ** for *P* < 0.001.

**Table S8** GO term enrichment in six clusters based on the PAM algorithm

| Cluster | BP | MF |
| --- | --- | --- |
| I | GO:0010200 response to chitin | – |
| II | GO:0006412 translation | GO:0003735 structural constituent of ribosome |
|  | GO:0042254 ribosome biogenesis | GO:0005200 structural constituent of cytoskeleton |
| III | GO:0009611 response to wounding | – |
| IV | GO:0009408 response to heat | – |
|  | GO:0009644 response to high light intensity |  |
|  | GO:0042542 response to hydrogen peroxide |  |
|  | GO:0010286 heat acclimation |  |
|  | GO:0006334 nucleosome assembly |  |
| V | GO:0006412 translation | GO:0003735 structural constituent of ribosome |
|  | GO:0042254 ribosome biogenesis |  |
|  | GO:0006414 translational elongation |  |
| VI | GO:0010200 response to chitin | GO:0003700 sequence-specific DNA binding transcription factor activity |
|  | GO:0006355 regulation of transcription, DNA-dependent |  |
|  | GO:0009751 response to salicylic acid stimulus |  |
|  | GO:0050832 defense response to fungus |  |
| VII | GO:0005507 copper ion binding | – |

*P*-value cutoff: 1.0E–04

BP: biological process

MF: molecular function

**References**

Akaike H (1974) A new look at the statistical model identification. *IEEE T Automat Contr*, **19**, 716–723.

Alexa A, Rahnenführer J, Lengauer T (2006) Improved scoring of functional groups from gene expression data by decorrelating GO graph structure. *Bioinformatics*, **22**, 1600–1607.

Ashburner M, Ball CA, Blake JA *et al*. (2000). Gene ontology: tool for the unification of biology. The Gene Ontology Consortium. *Nat Genet*, **25**, 25–29.

Cao D, Cheng H, Wu W, Soo HM & Peng J (2006) Gibberellin mobilizes distinct DELLA-dependent transcriptomes to regulate seed germination and floral development in Arabidopsis. *Plant Physiol*, **142**, 509–525.

Cao Y, Dai Y, Cui S, Ma L (2008) Histone H2B monoubiquitination in the chromatin of *FLOWERING LOCUS C* regulates flowering time in *Arabidopsis*. *Plant Cell*, **20**, 2586–2602.

Clough SJ, Bent AF (1998) Floral dip: a simplified method for *Agrobacterium*-mediated transformation of *Arabidopsis thaliana*. *Plant J*, **16**, 735–743.

Conesa A, Gotz S, Garcia-Gomez JM *et al.* (2005) Blast2GO: a universal tool for annotation, visualization and analysis in functional genomics research. *Bioinformatics*, **21**, 3674–3676.

Curtis MD, Grossniklaus U (2003) A gateway cloning vector set for high-throughput functional analysis of genes in planta. *Plant Physiol*, **133**, 462–469.

Eysteinsson T, Greenwood MS (1995) Flowering on long and short shoots of *Larix laricina* in response to differential timing of GA(4/7) applications. *Tree Physiol*, **15**, 467–469.

Fujiwara S, Oda A, Yoshida R *et al.* (2008) Circadian clock proteins LHY and CCA1 regulate SVP protein accumulation to control flowering in *Arabidopsis*. *Plant Cell*, **20**, 2960–2971.

Gould PD, Locke JC, Larue C *et al*. (2006) The molecular basis of temperature compensation in the *Arabidopsis* circadian clock. *Plant Cell*, **18**, 1177–1187.

Griffiths J, Murase K, Rieu I *et al*. (2006) Genetic characterization and functional analysis of the GID1 gibberellin receptors in *Arabidopsis*. *Plant Cell*, **18**, 3399–3414.

Gu X, Jiang D, Wang Y, Bachmair A, He Y (2009) Repression of the floral transition via histone H2B monoubiquitination. *Plant J*, **57**, 522–533.

Hanzawa Y, Money T, Bradley D (2005) A single amino acid converts a repressor to an activator of flowering. *Proc Natl Acad Sci U S A*, **102**, 7748–7753.

Harb A, Krishnan A, Ambavaram MM, Pereira A (2010) Molecular and physiological analysis of drought stress in Arabidopsis reveals early responses leading to acclimation in plant growth. *Plant Physiol*, **154**, 1254–1271.

Ichie T & Nakagawa M (2011) Dynamics of mineral nutrient storage for mast reproduction in the tropical emergent tree *Dryobalanops aromatica*. *Ecol Res* DOI: 10.1007/s11284–011–0836–1.

Igasaki T, Watanabe Y, Nishiguchi M, Kotoda N (2008) The *FLOWERING LOCUS T*/*TERMINAL FLOWER 1* family in Lombardy poplar. *Plant Cell Physiol*, **49**, 291–300.

Kardailsky I, Shukla VK, Ahn JH *et al*. (1999) Activation tagging of the floral inducer *FT*. *Science*, **286**, 1962–1965.

Kobayashi Y, Kaya H, Goto K, Iwabuchi M, Araki T (1999) A pair of related genes with antagonistic roles in mediating flowering signals. *Science*, **286**, 1960–1962.

Koornneef M, Hanhart CJ, van der Veen JH (1991) A genetic and physiological analysis of late flowering mutants in *Arabidopsis thaliana*. *Mol Gen Genet*, **229**, 57–66.

Kume T, Tanaka N, Kuraji K *et al*. (2011) Ten-year evapotranspiration estimates in a Bornean tropical rainforest. *Agr Forest Met*, **151**, 1183–1192.

Kumimoto RW, Zhang Y, Siefers N, Holt BF 3rd (2010). NF-YC3, NF-YC4 and NF-YC9 are required for CONSTANS-mediated, photoperiod-dependent flowering in *Arabidopsis thaliana*. *Plant J*, **63**, 379–391.

Lee JH, Yoo SJ, Park SH *et al*. (2007) Role of *SVP* in the control of flowering time by ambient temperature in *Arabidopsis*. *Genes Dev*, **21**, 397–402.

Litt A, Irish VF (2003) Duplication and diversification in the *APETALA1*/*FRUITFULL* floral homeotic gene lineage: implications for the evolution of floral development. *Genetics*, **165**, 821–833.

Ma S, Bohnert HJ (2007) Integration of *Arabidopsis thaliana* stress-related transcript profiles, promoter structures, and cell-specific expression. *Genome Biol*, **8**, R49.

Melzer S, Lens F, Gennen J, Vanneste S, Rohde A, Beeckman T (2008) Flowering-time genes modulate meristem determinacy and growth form in *Arabidopsis thaliana*. *Nat Genet*, **40**, 1489–1492.

Michaels SD, Amasino RM (1999) *FLOWERING LOCUS C* encodes a novel MADS domain protein that acts as a repressor of flowering. *Plant Cell*, **11**, 949–956.

Misson J, Raghothama KG, Jain A *et al*. (2005) A genome-wide transcriptional analysis using *Arabidopsis thaliana* Affymetrix gene chips determined plant responses to phosphate deprivation. *Proc Natl Acad Sci U S A*, **102**, 11934–11939.

Monteith JL, Unsworth MH (1990) *Principles of Environmental Physics*. 2nd edn. Edward Arnold, London.

Nemhauser JL, Hong F, Chory J (2006) Different plant hormones regulate similar processes through largely nonoverlapping transcriptional responses. *Cell*, **126**, 467–475.

Osuna D, Usadel B, Morcuende R *et al*. (2007) Temporal responses of transcripts, enzyme activities and metabolites after adding sucrose to carbon-deprived Arabidopsis seedlings. *Plant J*, **49**, 463–491.

Pelaz S, Gustafson-Brown C, Kohalmi SE, Crosby WL, Yanofsky MF (2001) *APETALA1* and *SEPALLATA3* interact to promote flower development. *Plant J*, **26**, 385–394.

Preston JC, Hileman LC (2010) SQUAMOSA-PROMOTER BINDING PROTEIN 1 initiates flowering in *Antirrhinum majus* through the activation of meristem identity genes. *Plant J*, **62**, 704–712.

Rhoades MW, Reinhart BJ, Lim LP *et al*. (2002). Prediction of plant microRNA targets. *Cell*, **110**, 513–520.

Sanger F, Air GM, Barrell BG *et al*. (1977) Nucleotide sequence of bacteriophage phi X174 DNA. *Nature*, **265**, 687–95.

Scheible WR, Morcuende R, Czechowski T *et al*. (2004) Genome-wide reprogramming of primary and secondary metabolism, protein synthesis, cellular growth processes, and the regulatory infrastructure of Arabidopsis in response to nitrogen. *Plant Physiol*, **136**, 2483–2499.

Schwab R, Palatnik JF, Riester M*, et al.* (2005) Specific effects of microRNAs on the plant transcriptome. *Dev. Cell*, **8**, 517–527.

Sheldon CC, Burn JE, Perez PP *et al*. (1999) The *FLF* MADS box gene: a repressor of flowering in Arabidopsis regulated by vernalization and methylation. *Plant Cell*, **11**, 445–458.

Tamura K, Peterson D, Peterson N, Stecher G, Nei M, Kumar S (2011) MEGA5: molecular evolutionary genetics analysis using maximum likelihood, evolutionary distance, and maximum parsimony methods. *Mol. Biol. Evol*, **28**, 2731–2739.

Thompson JD, Higgins DG, Gibson TJ (1994) Clustal-W: improving the sensitivity of progressive multiple sequence alignment through sequence weighting, position-specific gap penalties and weight matrix choice. *Nucleic Acids Res*, **22**, 4673–4680.

Wang JW, Czech B, Weigel D (2009) miR156-regulated SPL transcription factors define an endogenous flowering pathway in *Arabidopsis thaliana*. *Cell*, **138**, 738–749.

Wang JW, Park MY, Wang LJ *et al*. (2011) miRNA control of vegetative phase change in trees. *PLoS Genet*, **7**, e1002012.

Wilson RN, Heckman JW, Somerville CR (1992) Gibberellin is required for flowering in *Arabidopsis thaliana* under short days. *Plant Physiol*, **100**, 403–408.

Wu G, Park MY, Conway SR *et al*. (2009) The sequential action of miR156 and miR172 regulates developmental timing in *Arabidopsis*. *Cell*, **138**, 750–759.

Xu L, Ménard R, Berr A *et al*. (2009) The E2 ubiquitin-conjugating enzymes, AtUBC1 and AtUBC2, play redundant roles and are involved in activation of *FLC* expression and repression of flowering in *Arabidopsis thaliana*. *Plant J*, **57**, 279–288.

Yamaguchi A, Wu MF, Yang L, *et al*. (2009) The microRNA-regulated SBP-Box transcription factor SPL3 is a direct upstream activator of *LEAFY*, *FRUITFULL*, and *APETALA1*. *Dev. Cell*, **17**, 268–278.

Zhang JZ, Li ZM, Mei L, Yao JL, Hu CG (2009) *PtFLC* homolog from trifoliate orange (*Poncirus trifoliata*) is regulated by alternative splicing and experiences seasonal fluctuation in expression level. *Planta*, **229**, 847–859.
